# Can We Rationally Design and Operate Spatial Atomic Layer Deposition Systems for Steering the Growth Regime of Thin Films?

**DOI:** 10.1021/acs.jpcc.3c02262

**Published:** 2023-05-05

**Authors:** João Pedro Vale, Abderrahime Sekkat, Thomas Gheorghin, Semih Sevim, Eirini Mavromanolaki, Andreas D. Flouris, Salvador Pané, David Muñoz-Rojas, Josep Puigmartí-Luis, Tiago Sotto Mayor

**Affiliations:** † Transport Phenomena Research Centre (CEFT), Engineering Faculty of Porto University, Rua Dr Roberto Frias, 4200-465 Porto, Portugal; ‡ Associate Laboratory in Chemical Engineering (ALiCE), Engineering Faculty of Porto University, Rua Dr Roberto Frias, 4200-465 Porto, Portugal; § Université Grenoble Alpes, CNRS, Grenoble INP, LMGP, 38000 Grenoble, France; ∥ Laboratoire de Génie Chimique, Université de Toulouse, CNRS, 31013 Toulouse, France; ⊥ Multi-Scale Robotics Lab, Institute of Robotics and Intelligent Systems, ETH Zurich, Tannenstrasse 3, CH-8092 Zurich, Switzerland; # Creative Nano PC, 14451 Athens, Greece; ¶ Discovery Foundation, 70300 Heraklion, Crete, Greece; ∇ FAME Laboratory, Department of Physical Education and Sport Science, University of Thessaly, 38221 Volos, Greece; ○ Departament de Ciència Dels Materials i Química Física, Institut de Química Teòrica i Computacional, University of Barcelona (UB), 08028 Barcelona, Spain; ⧫ Institució Catalana de Recerca i Estudis Avançats (ICREA), Pg. Lluís Companys 23, 08010 Barcelona, Spain

## Abstract

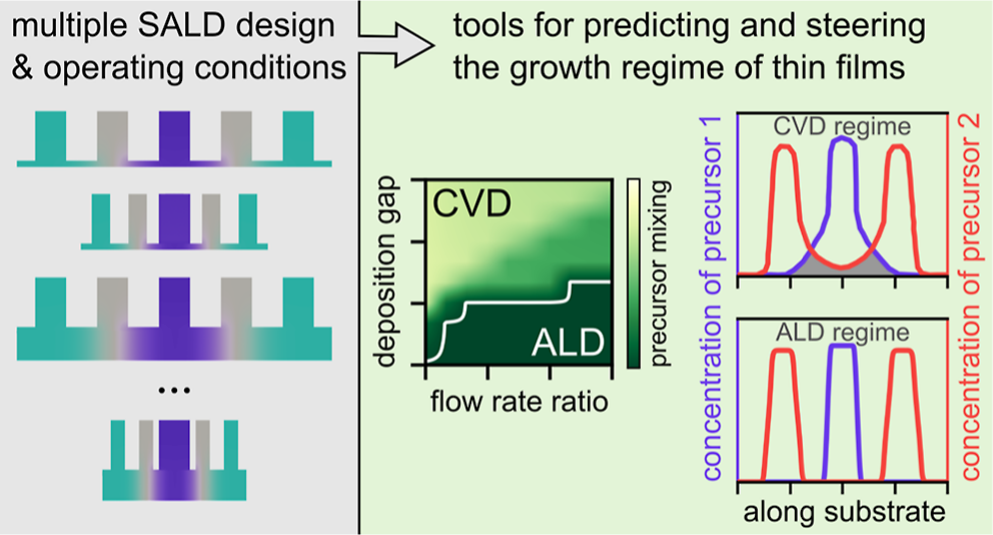

Fine control over the growth of materials is required to precisely tailor their properties. Spatial atomic layer deposition (SALD) is a thin-film deposition technique that has recently attracted attention because it allows producing thin films with a precise number of deposited layers, while being vacuum-free and much faster than conventional atomic layer deposition. SALD can be used to grow films in the atomic layer deposition or chemical vapor deposition regimes, depending on the extent of precursor intermixing. Precursor intermixing is strongly influenced by the SALD head design and operating conditions, both of which affect film growth in complex ways, making it difficult to predict the growth regime prior to depositions. Here, we used numerical simulation to systematically study how to rationally design and operate SALD systems for growing thin films in different growth regimes. We developed design maps and a predictive equation allowing us to predict the growth regime as a function of the design parameters and operation conditions. The predicted growth regimes match those observed in depositions performed for various conditions. The developed design maps and predictive equation empower researchers in designing, operating, and optimizing SALD systems, while offering a convenient way to screen deposition parameters, prior to experimentation.

## 1 Introduction

Progress in the fabrication of advanced micro- and nanodevices requires precise control over matter and a solid understanding of the underlying physical and chemical principles.^1^ For example, the ability to manipulate the number of molecular layers in a thin film of a functional material is valuable when producing functional materials, photovoltaic cells, light-emitting diodes, and micro/nanosensors, among others.^2−4^ Atomic layer deposition (ALD) is a fabrication technique that can be used to address this challenge, as it allows growing atomically precise and conformal thin films through the sequential exposure of substrates to precursors.^5−10^ In this layer-by-layer approach, a precursor gas initially reacts with a substrate and is then purged by an inert gas stream. A second precursor gas then reacts with the first precursor adsorbed on the substrate, after which it is purged, completing the deposition cycle. These substrate reactions are self-limiting, yielding one monolayer per cycle, and the purging steps prevent the mixing of precursors which would otherwise decrease the uniformity of the deposition.^11^ The deposition cycle is repeated until the required number of deposited layers is achieved. However, the need for long purge steps severely limits the throughput of ALD systems.^12^

**Figure 1:**
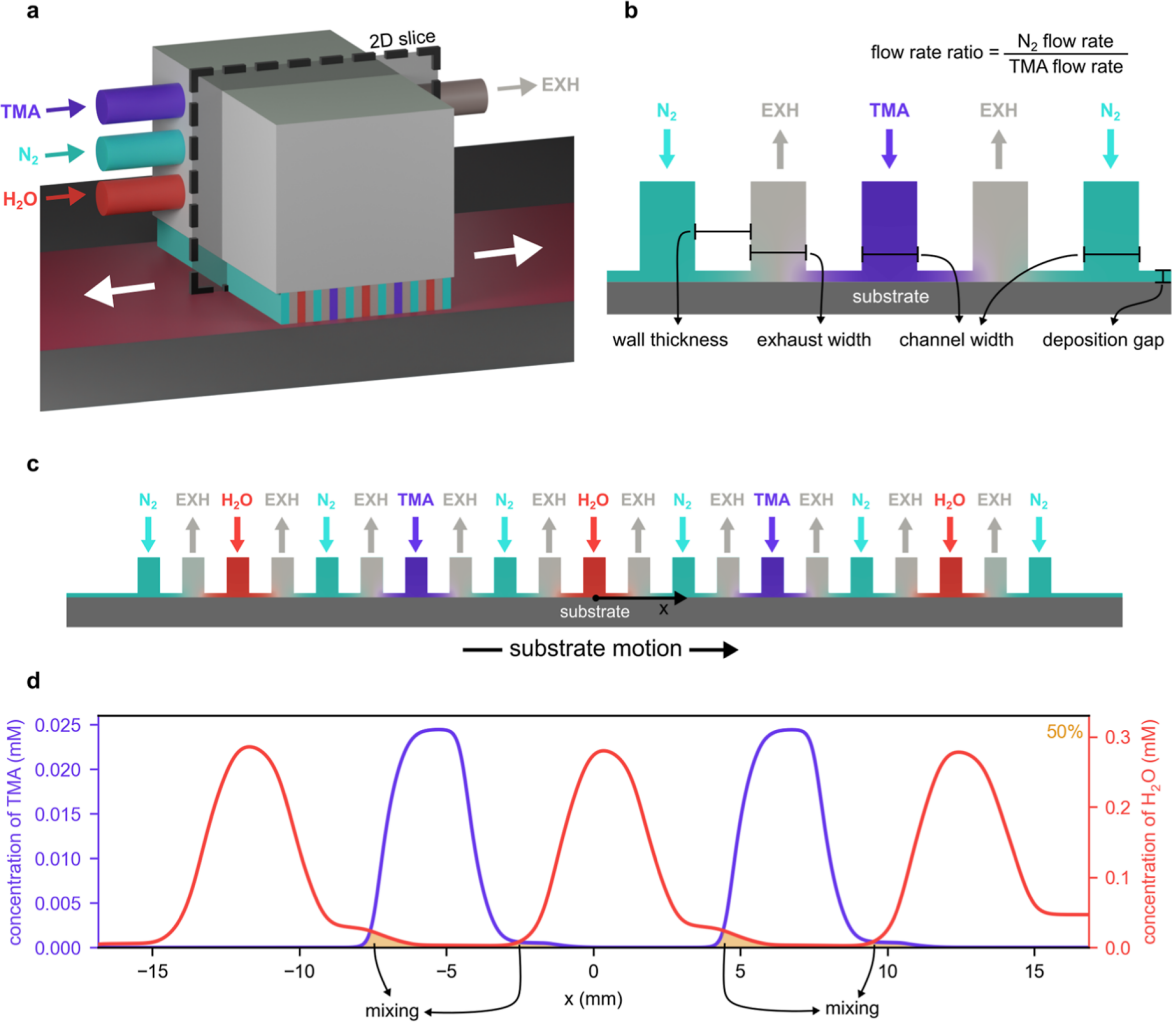
(a) 3D representation of the close-proximity SALD head considered in this study for the reaction between trimethylaluminum (TMA) and water (H_2_O). (b) Parameters that characterize the design and operation of the SALD head used in this study. (c) 2D domain considered in the simulations, representing a 2D slice of the 3D SALD head shown in (a). (d) Precursor concentration profiles along the substrate, for a set of conditions that result in intermixing of precursors. The orange area under the concentration profiles of TMA and H_2_O marks the proportion of the substrate with precursor intermixing, where precursors react with each other and the film grows by CVD (in this case, the proportion of the substrate with precursor intermixing corresponds to 50% of the substrate length, as shown in the top right corner of the graph).

Spatial ALD (SALD) is a variant of ALD featuring spatial, rather than temporal, separation of precursors,^13^ where purge steps are not needed, thus increasing the deposition rate by orders of magnitude compared to ALD (from ≈1 nm/min for ALD to ≈1 nm/s for SALD).^12−14^ In SALD, the precursor and inert gases are continuously flowed toward the substrate through a deposition head with multiple channels or slits (Figure 1a). The substrate is moved laterally relative to the deposition head, being sequentially exposed to the precursors during the movement. The inert gas introduced between the precursors (Figure 1a–c) serves as a curtain that prevents precursor intermixing and steers precursor molecules toward the exhausts. Moreover, the head is often placed in close proximity from the substrate, typically 20–200 μm, to further reduce and ideally prevent precursor intermixing.^15^

The extent of precursor intermixing is of crucial importance when using SALD, as it affects the uniformity of the deposited films.^15^ When precursors are well separated and thus react only with the substrate (or previously deposited layers) in a layer-by-layer manner, the deposition occurs in the ALD regime, characterized by an overall uniform and conformal film growth. In contrast, when there is significant mixing of precursors that can react with each other, the deposition occurs in the chemical vapor deposition (CVD) regime, resulting in faster but generally less uniform film growth.^16−18^ Although SALD systems are often used to deposit thin films in the ALD regime, they can also be used in the CVD regime, e.g., when aiming for higher throughput, when using precursors that do not react under ALD conditions, or to obtain films with different morphologies and properties.^17,19^ Furthermore, film thickness gradients, which are useful to quickly optimize the layer thickness of a device, can be achieved by depositing films in the CVD regime with a deposition head tilted relative to the substrate plane.^18^ Such gradients cannot be deposited in ALD because the absence of precursor mixing implies a film growth ultimately dictated by the thickness of the monolayer of the material being deposited. Therefore, depositing in different deposition regimes, either ALD or CVD, allows for the formation of films with very different physical and chemical properties, which may be of interest in different applications (e.g., ALD for the deposition of Al_2_O_3_ gas permeation barriers in flexible organic light-emitting diodes^20^ and to fabricate perovskite solar cells^21^ or CVD for rapid coating of functional ZnO nanoarrays^22^ and fast deposition of Mg-doped ZnO films in solar cells^23^).

Various parameters influence whether a deposition occurs in ALD or CVD regimes when using SALD heads: operational parameters such as the deposition gap and the chosen flow rates and head design parameters such as the width of the gas channels and the thickness of the channel walls.^13^ Importantly, optimizing the design of the SALD head is particularly promising for future developments, given the ease of customizing SALD heads via rapid prototyping techniques, e.g., 3D-printing.^17,24^

Some of the parameters that can be leveraged to minimize the mixing of precursors during deposition of thin films have been identified. For instance, decreasing the gap between the substrate and the deposition head and increasing the flow rate of the separation gas have been shown to prevent precursor mixing in SALD systems for porous,^25^ nonporous,^26−29^ and microgroove^30^ substrates. The application of a slight vacuum at the exhausts, which makes them more efficient at purging the precursors, is also known to decrease precursor intermixing,^26,31^ although some deposition head designs can operate well without vacuum at the exhaust.^32^ Moreover, several studies suggest that moving the head slowly relative to the substrate generates high-quality films by not dragging precursors from one region to the other during movement.^26,28^ On this topic, Pan used density functional theory combined with computational fluid dynamics to simulate the flow and mass transfer in SALD and found that although the substrate movement in relation to the deposition head affects the gas flow, it does not significantly disturb the separation of precursors, even at high velocities (1.5 m/s).^33^ Finally, using a dynamic mesh method, Cong and colleagues showed that increasing the distance between gas injectors could be more effective at preventing precursor intermixing than increasing the flow rate of the separation gas, highlighting the important role of the head design in the deposition performance.^28^

Although the above contributions provide qualitative information on the influence of some parameters of SALD systems over the film growth regime, the existing literature is still insufficient to predict whether film growth will occur by ALD or CVD when using a specific set of conditions. The lack of quantitative information makes it necessary to perform laborious trial-and-error experimentation to identify the conditions and head designs that result in the sought growth regimes (ALD or CVD), which is time-consuming, unpractical, and costly. Furthermore, given that 3D printing widens the possibilities in terms of design and customization of SALD heads, a complete understanding of the influence of the SALD head design on the separation of precursors is needed to enable further optimization of this promising process.

Here, we report on a series of numerical simulation studies that quantitatively inform on how to design and operate close-proximity SALD heads to achieve the desired thin-film growth regime (ALD or CVD). First, we investigated the influence of multiple parameters of the head design and operation conditions over the precursor intermixing and the associated thin-film growth. Then, we have built design maps of the proportion of the substrate with precursor intermixing (i.e., regions where film growth occurs by CVD), as a function of the head design parameters and the chosen operating conditions. The generated numerical data were then used to develop a predictive equation allowing us to predict the thin-film growth regime depending on the chosen head design parameters and operating conditions, whose predictions were compared to our experimental results. The developed design maps and predictive equation help users in designing, operating, and optimizing SALD systems, for deposition in the targeted growth regimes (ALD or CVD), without the need for time-consuming trial-and-error experimentation.

## 2 Methods

### 2.1 Problem Formulation

A close-proximity SALD head typically consists of multiple parallel channels that move gas precursors toward the substrate, where they react before being purged through exhausts. In this head configuration, nitrogen gas (N_2_) serves both as the gas carrier to move precursors toward the substrate and as the gas curtain to separate the precursors. In the present study, the deposition of aluminum oxide (Al_2_O_3_) using TMA and H_2_O as precursors, a common model reaction in ALD studies, was assumed to be done with a moving SALD head at 200 °C.^8,34−38^ We considered a deposition head used in previous studies^39,40^ (Figure 1a), which features two channels for the metallic precursor (TMA) and three channels for the oxygen precursor (H_2_O), assuring the deposition of two cycles per pass. Additionally, the deposition head contains multiple inert gas (N_2_) and exhaust (EXH) channels to prevent the mixing of precursors (Figure 1b,c). The transport processes influencing the precursor intermixing depend on the geometric features of the head, i.e., wall thickness (wall_thick_) and exhaust width (exh_width_), and the chosen operating conditions, i.e., deposition gap (dep_gap_), precursor flow rate (pfr), and flow rate ratio (frr), as illustrated in Figure 1b. For this reason, the values of these parameters were changed in a systematic way to study their influence on the flow and mass transport during deposition. Note that throughout this study, the precursor flow rate refers to the flow rate of the TMA or H_2_O stream after dilution with the carrier gas (TMA + carrier, or H_2_O + carrier), and the flow rate ratio refers to the ratio between the flow rate of the separation gas and that of the precursor.

Depending on the deposition head design and operation parameters, precursors passing through close-proximity SALD heads may mix and react with each other (CVD regime), rather than reacting only with the substrate or previously deposited layers (ALD regime). To quantify the influence of the above parameters on precursor intermixing, we calculated the precursor intermixing proportion at the substrate, as the proportion of the substrate under the deposition head where there is significant mixing of precursors. This was calculated by dividing the length of the substrate exposed to both precursors at the same time (i.e., orange regions in Figure 1d) by the total length of the head (along the *x*-axis in Figure 1c). The precursor mixing proportion thus varies between 0 and 100%. A precursor intermixing proportion of 0% indicates no mixing of precursors throughout the entire substrate, thus film growth occurring in the ALD regime, whereas a precursor mixing proportion of 100% indicates that the entire substrate is exposed to both precursors and, thus, experience film growth in the CVD regime.

### 2.2 Modeling Assumptions and Boundary Conditions

The deposition head was modeled in 2D because its thickness (5 cm) is much larger than the width of the channels (500 μm, Figure 1a), which reduces the influence of the thickness in the transport processes inside the head. The flow in the head was assumed to be laminar, given the associated low Reynolds number (1 < *Re* < 6). The fluid properties (density of 0.71 kg·m^–3^ and dynamic viscosity of 2.52 × 10^–5^ kg·m^–1^·s^–1^)^41,42^ were assumed to be those of N_2_ at 200 °C as the precursors were diluted in N_2_, thus having little impact on the properties of the mixture. The diffusion coefficients of TMA and H_2_O at 200 °C were assumed to be 1.75 × 10^–5^ and 5.53 × 10^–5^ m^2^·s^–1^, respectively.^43^

In experiments with a similar deposition head, saturated currents of precursors were generated by flowing the carrier gas through bubblers,^44,45^ which were then diluted before being delivered to the head (dilution ratios: 15 sccm of saturated TMA in 250 and 150 sccm of saturated H_2_O in 375 sccm).^46−48^ Therefore, in the simulations, the partial pressures of TMA and H_2_O at the precursor inlet boundaries (Figure 1b) were assumed to be 100 and 1250 Pa, respectively, based on their saturated partial vapor pressure for 25 °C (temperature of the bubblers) obtained via the Antoine equation and the dilution of the precursor lines. Note that the values of precursor flow rate, which vary between 75 and 300 sccm (cm^3^/min at standard conditions), correspond to the sum of the TMA/H_2_O flow rate and carrier gas flow rate in each individual channel (therefore, the total flow rate entering the head is the precursor flow rate multiplied by the number of channels). Furthermore, after correction based on the temperature of the process (200 °C), the precursor flow rate in each precursor channel considered in the simulations varied between 2 × 10^–6^ and 8 × 10^–6^ m^3^/s because of the change in gas density.

The outlet boundary conditions at the exhausts and at the sides of the gap were assumed to be atmospheric pressure,^14^ and no-slip boundary conditions were considered at the walls of the head and at the substrate. Preliminary simulation results suggested that, depending on the SALD design and operating conditions, there may be a difference between the concentration curves of precursors for static and dynamic depositions (see Figure S1). Therefore, we assumed that the substrate was moving relative to the SALD head at 100 mm s^–1^, a typical scanning speed in SALD systems.^32,40,49,50^ This motion was introduced in the model as a moving wall boundary condition, which implies that the fluid elements in contact with the substrate are moving with it at the same velocity (due to the no-slip condition considered).

We assumed that the CVD regime occurs when there is non-negligible precursor intermixing at the substrate, and this requires the definition of the minimum partial pressures that are non-negligible. When examining the concentrations and partial pressures predicted in our simulations together with the films obtained experimentally, we found that acknowledging intermixing when there was at least 0.1 Pa of TMA and 1.25 Pa of H_2_O (corresponding to 0.1% of the partial vapor pressures entering the deposition head for both precursors) would ensure full consistency between the predicted and the experimentally observed growth regimes. This suggests that these minimum partial pressures can be used to identify precursor intermixing and onset of CVD growth (see Section 2.4 for further details). Thus, when the partial pressure of at least one of the precursors was below the mentioned partial pressures along the entire substrate length, the deposition was considered to occur in the ALD regime, and the precursor intermixing proportion in the substrate was considered to be 0%.

### 2.3 Numerical Methods

The flow and mass transport in the SALD heads were simulated using a computational fluid dynamics approach based on finite volume modeling. Velocity, pressure, and species concentration were calculated by coupling the Navier–Stokes equation for an incompressible Newtonian fluid, the continuity equation, and the species transport equation, which were, respectively, given by
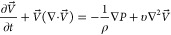
1
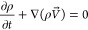
2
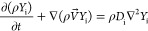
3where *V⃗* is the velocity vector, ∇ is the divergence operator, ρ is the fluid density, *P* is the pressure, υ is the kinematic viscosity, ∇^2^ is the Laplacian operator, *Y*_i_ is the mass fraction of species I, and *D*_i_ is the diffusion coefficient of species i. A steady-state, double-precision, pressure-based solver was used, considering second-order discretization, and the SIMPLE algorithm was used for velocity–pressure coupling.

Simulations were performed using a mesh containing around 6 × 10^4^ cells (30 cells distributed along the width and length of the channels, the walls, and the gap, as seen in Figure S2), which was found to produce mesh-independent results at low computational cost (Figure S3). A convergence criterion (which measures the residuals of each quantity, scaled by the flow rate through the domain) of 10^–4^ was considered, as results did not differ when stricter criteria were used (Figure S3).

### 2.4 Validation of Model Predictions against Published and New Experimental Data

For validation purposes, the velocity profile inside precursor channels was obtained using the present numerical approach and compared to an analytical solution of the velocity inside rectangular channels^51^ (Figure S4). Additionally, static and dynamic concentration profiles of the precursor along the substrate were obtained using the present numerical approach and compared to concentration profiles reported recently for a SALD head similar to that of the present study (Figure S5).^52^

To confirm that the results obtained with the present numerical approach match the thin-film growth regime that is observed in different experimental conditions, we numerically replicated eight different experiments (Table S2): four depositions reported in the literature^18^ and another four depositions performed in this work. Our depositions were performed using a SALD head with a channel width of 500 μm, wall thickness of 800 μm, exhaust width of 500 μm, deposition gaps of 30, 90, and 300 μm, precursor flow rate of 125 sccm, and flow rate ratio from 1 to 2. TMA and H_2_O were used as precursors, and the silicon wafer substrate was kept at 200 °C. Because these depositions were performed in the static mode (which prevents the full ALD cycle), no visible film growth was expected when depositing in the ALD regime. Therefore, visible film growth was expected only when depositing in the CVD regime.

Figures S4–S6 and Table S2 show the comparison of the numerical predictions generated with our simulation model and the data present in the literature and/or obtained experimentally in this work. The consistency and agreement between the numerical predictions and the literature/experimental data confirm the adequacy of the modeling and numerical approach followed in this work and therefore indicate that the present model can be used to predict the flow and the mass transport in SALD heads, as well as the film growth regimes to be expected in different operating conditions.

### 2.5 Cases Considered in Numerical Simulations

To study how to optimize SALD heads for controlling the film growth regime, we considered variations in the head geometric parameters (i.e., exhaust width and wall thickness; Figure 1b) and the operating conditions (i.e., deposition gap, precursor flow rate, and flow rate ratio; Figure 1b). We considered wall thicknesses of 50–1000 μm, exhaust widths of 100–500 μm, deposition gaps of 50–500 μm, precursor flow rates of 75–300 sccm, and flow rate ratios of 1–10, for a channel width of 500 μm (Table 1). These ranges were chosen to encompass the values that can be used experimentally for each parameter,^15,30,32,52^ with fabrication limitations guiding the choice of the minimum wall thickness (50 μm). The values considered for each parameter of interest (Table 1) were combined following a full factorial design-of-experiments approach, where all the possible combinations between the different parameters generated independent simulation cases. This resulted in a total of 2700 simulation cases (i.e., 3 wall thicknesses × 3 exhaust widths × 10 deposition gaps × 10 flow rate ratios × 3 precursor flow rates), which allowed us to analyze the influence of each parameter and their combinatory effect over the precursor intermixing.

**Table 1:** Parameters Considered in the Study, Which Were Combined Following a Full Factorial Design-of-Experiments Approach to Generate the 2700 Simulation Cases Considered^a^

wall thickness (μm)	exhaust width (μm)	deposition gap (μm)	precursor flow rate (sccm)	flow rate ratio (-)
1000	500	500	75	1
500	250	450	150	2
50	100	400	300	3
		350		4
		300		5
		250		6
		200		7
		150		8
		100		9
		50		10

a The precursor flow rate corresponds to the sum of the TMA/H_2_O and carrier gas flows, and the flow rate ratio is the ratio between the flow rate of separation gas and that of the precursor. The channel width in all simulations was 500 μm.

## 3 Results and Discussion

### 3.1 Influence of Parameters on Precursor Intermixing

**Figure 2:**
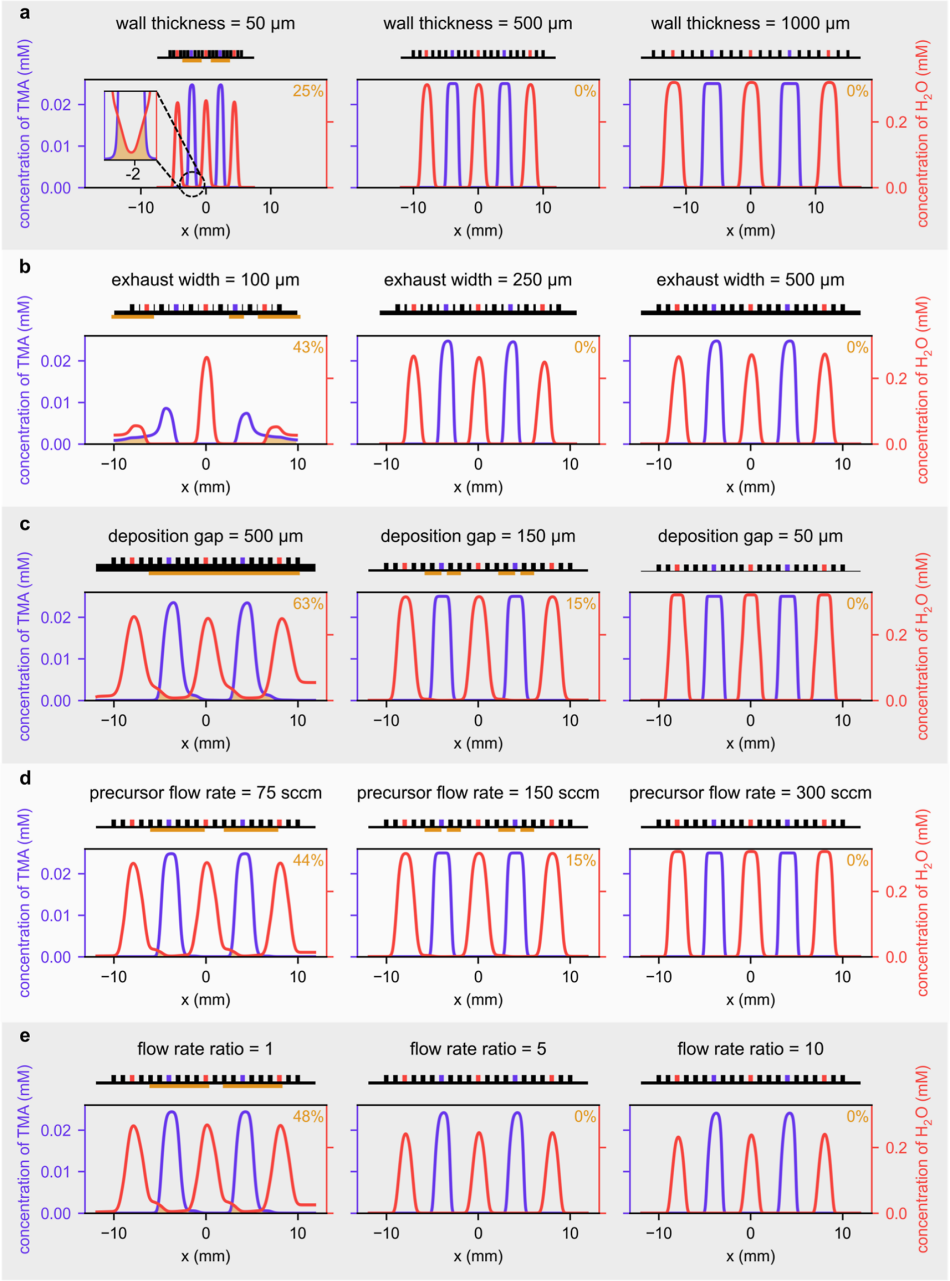
Concentration profile of TMA and H_2_O along the substrate (*x*-axis) while varying one parameter. (a) Wall thicknesses from 50 to 1000 μm, for a deposition gap of 150 μm, exhaust width of 500 μm, precursor flow rate of 150 sccm, and flow rate ratio of 2. (b) Exhaust widths from 100 to 500 μm, for a wall thickness of 500 μm, deposition gap of 300 μm, precursor flow rate of 150 sccm, and flow rate ratio of 5. (c) Deposition gaps from 500 to 50 μm, for a wall thickness of 500 μm, exhaust width of 500 μm, precursor flow rate of 150 sccm, and flow rate ratio of 1. (d) Precursor flow rates from 75 to 300 sccm, for a wall thickness of 500 μm, deposition gap of 150 μm, exhaust width of 500 μm, and flow rate ratio of 1. (e) Flow rate ratios from 1 to 10, for a wall thickness of 500 μm, deposition gap of 200 μm, exhaust width of 500 μm, and precursor flow rate of 75 sccm. The specific constant parameters considered in (a–e) were selected because they result in concentration profiles that better illustrate the effect of each parameter under analysis. Qualitatively similar effects would be obtained for other sets of constant parameters. Illustrations of the deposition heads of each simulation case are shown above each plot together with orange line marking where precursor intermixing is expected. In each simulation case, the orange areas under the concentration profiles mark the regions with precursor intermixing, and the specific proportion of the substrate with precursor intermixing is shown in orange at the plots’ top-right corner.

We started by studying the influence of the head geometric features (i.e., wall thickness and exhaust width) and operating conditions (i.e., deposition gap, precursor flow rate, and flow rate ratio) on precursor intermixing. The effect of each parameter over the concentration profile of the precursors along the substrate is shown in Figure 2, where the parameters of interest (e.g., wall thickness in Figure 2a) were varied, for constant values of all the other parameters. Precursor intermixing is highlighted in orange, and the proportion of the substrate with precursor intermixing is shown at the top right corner of each plot. Illustrations of the deposition heads are shown above each plot.

Increasing the thickness of the walls between consecutive channels decreases precursor intermixing,^28^ due to the larger distance that precursor molecules have to travel to mix (Figure 2a). For instance, an increase in the thickness of the walls from 50 to 1000 μm reduced the proportion of the substrate with precursor intermixing from 25 to 0% (Figure 2a), effectively preventing CVD film growth during the deposition. When aiming at ALD film growth, the walls of the deposition heads must be sufficiently thick to ensure that precursors are well separated and there is no overlap between the concentration profiles of the different precursors. Yet, note that thicker walls will imply larger deposition heads, which, if moved at a constant scanning speed, will lead to lower deposition rates (as each passage of larger heads takes longer). In contrast, heads with thinner walls (e.g., 50 μm, Figure 2a) lead to concentration profiles with lower maximum values, which may require lower scanning speeds to ensure substrate saturation. Additionally, thermal deformation may occur in SALD heads fabricated with thin walls (e.g., 50 μm), particularly when employing plastic materials.^17^ Therefore, the wall thickness of a deposition head should be optimized based on the target film growth regime, the sought deposition rate, and the fabrication limitations.

The effect of decreasing the width of the exhaust channels (Figure 2b) is somewhat similar to that of reducing the thickness of the walls between the different channels (Figure 2a). Both effects lead to narrower concentration profiles and increasing precursor intermixing, due to the decreasing distance between consecutive precursor channels. However, there is a limit to how small the exhaust channels may be, as they have to be large enough to accommodate both the precursor and the inert currents, which otherwise may escape outward along the substrate, causing precursor outflow through the sides of the gap (Figure 2b, exhaust width of 100 μm). When that occurs, the precursor concentration profiles will show non-negligible concentrations for low and high values of the *x* coordinate and, consequently, large proportions of the substrate with precursor intermixing.

Furthermore, when the exhausts are narrower, the larger outflow through the sides of the gap reduces the velocities in the nearby exhausts relative to those at the center of the head. This allows for more transport by diffusion near the sides of the head, resulting in wider concentration profiles and lower concentrations of precursors. Therefore, although decreasing the width of the exhaust channels may be a way to decrease the overall size of the deposition head and therefore increase the deposition rate, careful analysis is necessary to ensure that the exhausts operate efficiently, and the head does not lead to precursor outflow nor precursor intermixing, which could ultimately lead to uncontrolled deposition through the sides of the gap and/or significant film growth by CVD.

Decreasing the deposition gap between the head and substrate from 500 to 50 μm reduced the proportion of the substrate with precursor intermixing from 63 to 0% (Figure 2c) in line with previous results.^32^ Smaller gaps imply higher flow velocities and, thus, lower diffusion times (molecules moving at higher velocity have less time to diffuse before exiting through the exhausts/sides of the head). This reduces the diffusion and mixing of precursors, with their concentration profiles overlapping less (orange region in Figure 2c). Conveniently, smaller deposition gaps imply concentration profiles that have higher maximum concentration (Figure 2c) which could lead to faster reactions with the substrate and could enable the deposition of materials from precursors with low reactivity and/or low volatility. Additionally, the fact that smaller deposition gaps result in higher precursor concentrations and faster reactions indicates that the saturation of the substrate can be faster and, thus, may be compatible with higher scanning speeds. The deposition gap can be easily tuned in most experimental setups, and, therefore, adjusting it can be the most practical way of dictating whether film growth occurs in CVD or in ALD regimes. However, it may be challenging to perform depositions at very small gaps (<100 μm), especially when controlling the gap manually, because undesirable tilting of the SALD head and scraping the substrate can occur.^31^ Therefore, the multitude of effects associated to a change in the deposition gap highlights the importance of adequate design, operation, and optimization of SALD systems, to find the optimal trade-offs.

Increasing the precursor flow rate from 75 to 300 sccm for a constant flow rate ratio decreased the transport by diffusion because of the shorter diffusion time (i.e., the time over which the precursor molecules can diffuse). Because of this, the precursor concentration profiles became narrower (Figure 2d, from left to right), and the proportion of substrate with precursor intermixing decreased from 44 to 0% (orange region in Figure 2d). Thus, the precursor flow rate must be tuned to guarantee that the deposition occurs in the targeted film growth regime. Additionally, when choosing the precursor flow rate, one should also consider that low flow rates may lead to depletion of the precursor near the substrate during deposition, whereas high flow rates may generate excessive pressures and/or waste of reagents, which has economic and environmental consequences.^53,54^ Therefore, a careful selection of the precursor gas flow rate is key not only to control the film growth regime but also to ensure that depositions are of high quality and cost-efficient.

Increasing the flow rate ratio (Figure 2e) for constant precursor flow rate decreased the overlap between the two precursor concentration curves and allowed us to go from film growth by CVD (Figure 2e, FRR = 1) in 48% of the substrate length to film growth by ALD (Figure 2e, FRR = 5 and FRR = 10) in the entire substrate. The fact that increasing the flow rate ratio reduced the precursor intermixing is consistent with previous results^29^ and is the consequence of the increasing separation of precursors induced by the increasing flow rate of inert gas relative to those of the precursors. However, it is important to note that increasing the flow rate ratio also led to a decrease in the maximum concentration of precursors near the substrate, because the higher dilution of the precursors inside the gap reduced the diffusive transport due to the lower diffusion times associated with the larger velocities. These lower maximum concentrations at higher flow rate ratios could elicit slower reactions with the substrate, which can negatively impact the deposition rate, and may require the use of lower scanning speeds to ensure saturation of the substrate. Furthermore, low flow rate ratios may be preferable, for example, when depositing at low temperatures, depositing materials with low-reactivity precursors, or coating high-aspect-ratio features^55^ because of the associated higher concentration of precursors near the substrate. Therefore, the flow rate ratio to use in a SALD system must be chosen considering the trade-offs between the different effects that influence the extent of precursor intermixing and the required precursor concentration.

Based on the above, the extent of precursor intermixing, and thus of film growth by CVD, can be reduced by (1) increasing the thickness of the walls, (2) increasing the width of the exhaust channels, (3) decreasing the deposition gap, (4) increasing the flow rate of the precursors, and (5) increasing the flow rate ratio. Each parameter affects multiple aspects of the deposition process differently and must, thus, be carefully considered based on the deposition requirements such as the targeted film growth regime, acceptable extent of precursor intermixing, sought scanning speed, and cost, among others. This is of paramount importance to enable the use of SALD as a high-throughput and flexible patterning technique.

### 3.2 Design Maps to Optimize SALD Head Design and Operating Conditions for Film Growth in ALD or CVD

**Figure 3:**
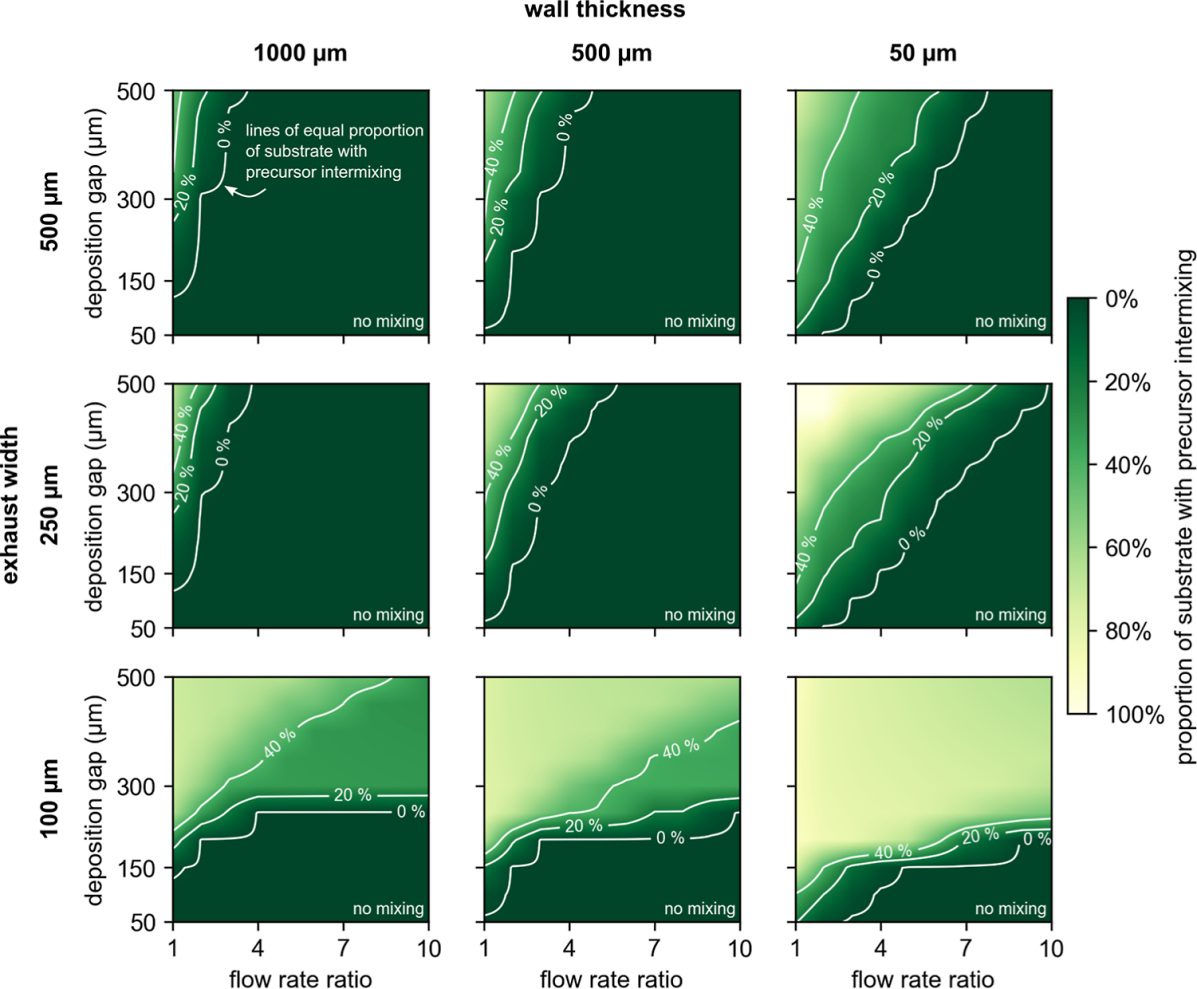
Design maps of the proportion of the substrate with precursor intermixing as a function of deposition gap and flow rate ratio, for wall thicknesses of 1000, 500, and 50 μm (columns) and exhaust widths of 500, 250, and 100 μm (rows), for a precursor flow rate of 150 sccm. White lines in the maps identify the conditions of equal precursor intermixing proportion. The region below the 0% line represents the conditions that lead to null precursor mixing and, thus, film growth by ALD. The region above the 0% line represents the conditions that lead to some precursor mixing and, thus, film growth by CVD. The specific value of the precursor intermixing proportion indicates the proportion of substrate length that is exposed to film growth by CVD. The design maps in this figure show the results of 900 different simulation cases.

Given the importance of the head design parameters and operating conditions over the extent of precursor intermixing (Section 3.1) and the associated film growth by CVD or ALD, it is crucial to offer users of SALD systems detailed information on the film growth regime to be expected under different conditions. To this end, we prepared a series of 27 design maps informing on the proportion of the substrate with precursor intermixing (Figures 3, and S7 and S8), as a function of the head design parameters (i.e., wall thickness and exhaust width) and the operating conditions of the deposition (i.e., deposition gap, precursor flow rate, and flow rate ratio). These maps compile the results of the 2700 simulation cases analyzed in this study and enable researchers and SALD users to design the heads and choose the suitable operating conditions, based on their targeted film growth regime, before starting time-consuming and costly rounds of experimental testing.

Each design map in Figure 3 shows the proportion of the substrate with precursor intermixing zone, for 100 different simulation cases, obtained for a range of deposition gaps (50–500 μm) and flow rate ratios (1–10), for constant values of exhaust width (100–500 μm) and wall thickness (50–1000 μm), and for a precursor flow rate of 150 sccm (a typical flow rate in SALD works; design maps for precursor flow rates of 75 and 300 sccm are given in Figures S7 and S8, respectively). In each map, the white lines identify the conditions of equal proportion of substrates with precursor intermixing, with the region below the 0% line representing the conditions that lead to null precursor mixing and, thus, film growth by ALD and the region above the 0% line representing the conditions that lead to some precursor mixing and, thus, film growth by CVD. The maps in Figure 3 allow identifying the head design parameters and operating conditions that favor film growth by either ALD or CVD, offering a way to optimize the head design and operating conditions based on the deposition requirements. For example, a user looking to deposit a film in the ALD regime will see in Figure 3 that a deposition head with a wall thickness of 500 μm and an exhaust width of 500 μm (upper center plot in Figure 3), operated with a precursor gas flow rate of 150 sccm, flow rate ratios of 4–10, and a deposition gap of 150 μm, should produce the sought ALD film growth. If the same user would like to increase the deposition rate by allowing film growth by CVD, they would see that this can be achieved, for example, by decreasing the flow rate ratio to 1 or by increasing the deposition gap to 500 μm for a flow rate ratio of 3 (see the upper center map in Figure 3). Moreover, the same head operated with a deposition gap of 300 μm, a flow rate ratio of 3, and a precursor flow rate of 75 sccm (upper center map in Figure S7) should also lead to CVD film growth. The maps in Figures 3 and S7 and S8 offer, before any experimental trial-and-error procedure, specific and objective data on the expected proportion of the substrate with precursor intermixing, greatly accelerating the process of designing and planning the use of SALD systems to deposit in the targeted film growth regimes.

### 3.3 Data-Driven Model to Enable Predicting the Film Growth Regime

The design maps discussed in the previous section aggregate the information of 2700 simulation cases and offer quantitative data on how to design and operate SALD heads, for a wide set of design and operating conditions. However, because the developed design maps were obtained for heads with specific wall thicknesses (50, 500, and 1000 μm) and exhaust widths (100, 250, and 500 μm; Table 1), we have used data-driven modeling^56^ to develop an equation that can predict the film growth regime, for any set of design and operation parameters, within the ranges of parameters considered in this study. To this end, using a binary classification approach, the 2700 simulation cases (Table 1) were classified through a variable regime_expected_ as corresponding to depositions occurring in ALD when the proportion of the substrate with intermixing was 0% in the numerical simulations (regime_expected_ = ALD) or as corresponding to depositions occurring in CVD when the proportion of the substrate with intermixing was >0% in the numerical simulations (regime_expected_ = CVD). Then, we used the least-squares method^57^ to develop the following equation
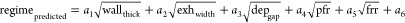
4that fits the regime_expected_ data and enables computing a variable regime_predicted_ representing the predicted film growth regime, as a function of the different design and operation parameters (i.e., wall thickness, exhaust width, deposition gap, precursor flow rate, and flow rate ratio in eq 4). The variables *a*_1_ to *a*_6_ are the coefficients of the different terms in the equation, and their values and standard errors, given in Table 2 (see the Supporting Information for details on other predictive models compared in this work), were obtained using the least-squares method.^57^

**Table 2:** Values and Standard Errors for Each Coefficient Used in the Predictive Equation Used in the Present Study (Eq 4)^a^

parameter	coefficient	value	standard error
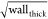	*a*_1_	–1.28 × 10^–2^	6.18 × 10^–4^
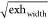	*a*_2_	–2.92 × 10^–2^	1.24 × 10^–3^
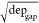	*a*_3_	4.57 × 10^–2^	1.32 × 10^–3^
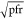	*a*_4_	–3.34 × 10^–2^	1.76 × 10^–3^
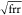	*a*_5_	–0.30	9.32 × 10^–3^
1	*a*_6_	1.52	4.44 × 10^–2^

a The coefficients characterize the effect of each parameter on the film growth regime. The coefficients were obtained considering the units in Table 1, but different units can be used if the coefficients are adjusted accordingly. Wall thickness = wall_thick_, exhaust width = exh_width_, deposition gap = dep_gap_, precursor flow rate = pfr, and flow rate ratio = frr. The coefficient *a*_6_ is the constant term in eq 4.

Having developed eq 4 to predict the film growth regime, we then defined a threshold for the variable regime_predicted_ below which the depositions are predicted to occur in ALD and above which the depositions are predicted to occur in CVD. The threshold allows translating the numeric result of eq 4 (regime_predicted_) into a binary classification of film growth regime, ALD or CVD, which can be directly compared to the regime expected based on the numerical simulations (regime_expected_). A threshold of 0.54 was found adequate for eq 4 because it ensured that most of the cases in the ALD regime were correctly classified, while only ≈10% of the cases in the CVD regime were incorrectly classified (Figure S9 and Table S3).

To characterize the performance of the developed predictive equation, we calculated its accuracy (i.e., percentage of cases correctly predicted to correspond to depositions in ALD or CVD) and its precision (i.e., percentage of cases correctly predicted to correspond to depositions in ALD relative to those correctly and incorrectly predicted to correspond to depositions in ALD; see the Supporting Information for details). Note that an additional set of 540 numerical simulations was used for computing the equation accuracy and precision to ensure that the evaluation of the performance of the predictive equation is not biased and that the equation can correctly classify data that were not used to develop it. When used with a threshold of 0.54, the developed predictive equation was found to have an accuracy of 92.2% and a precision of 96.0%, two indicators of robust performance at classifying the film growth regime. More importantly, the set of experimental depositions performed in the present study (#1–#4, in Table S2) confirmed the film growth predictions obtained with the developed predictive equation. The high accuracy and precision of the predictive eq 4, and the fact that it correctly predicted the film growth regimes obtained experimentally in this work, show that the equation can be used for predicting the film growth regime to be expected when depositing with different head designs and operating conditions.

**Figure 4:**
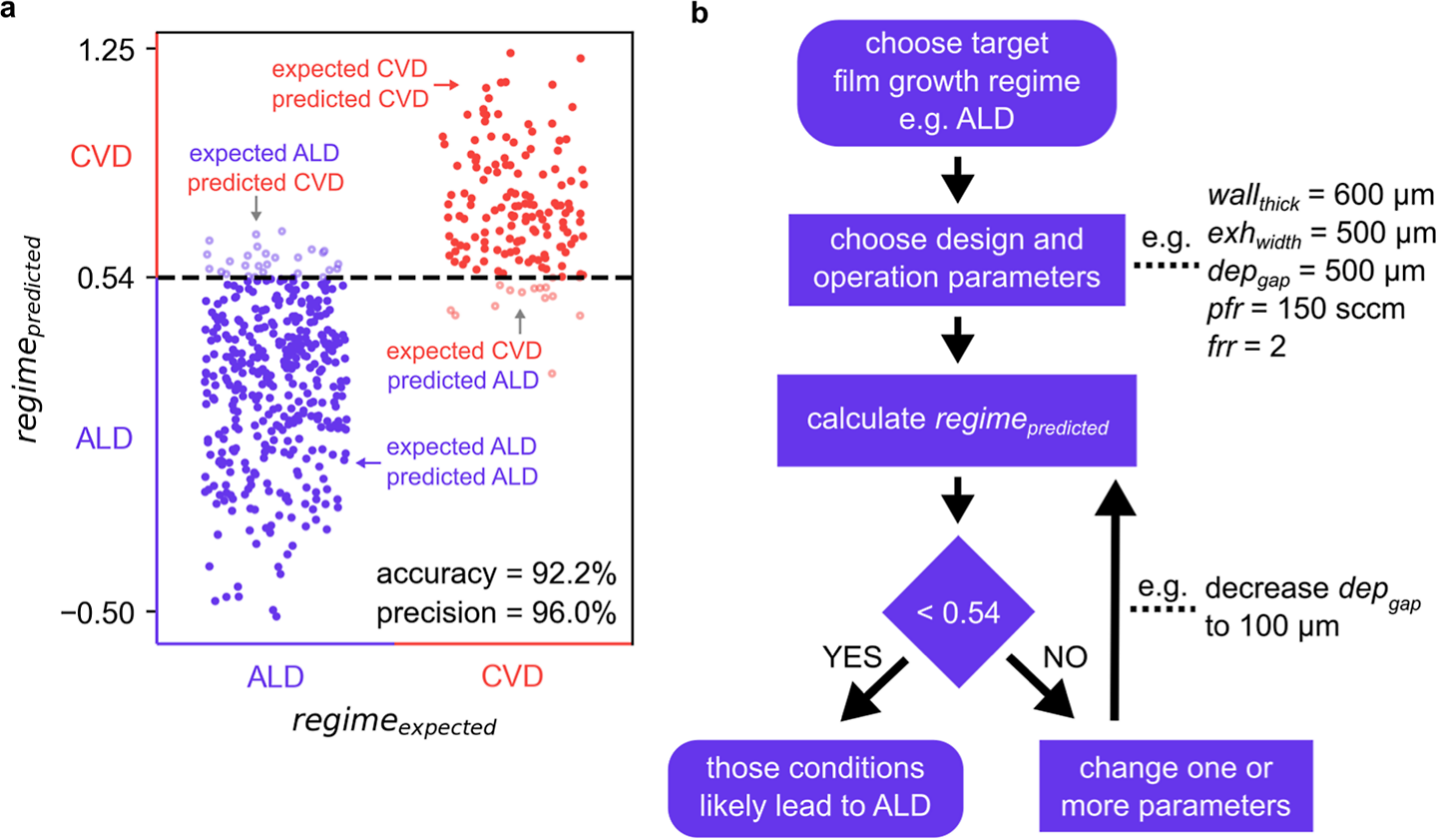
(a) Comparison between the growth regime predicted by the developed predictive equation (regime_predicted_) and the growth regime expected based on the numerical simulation (regime_expected_), for an additional set of 540 simulation cases that were conducted for testing. Cases below the dashed line (chosen threshold) are predicted to lead to depositions in the ALD regime. Purple dots mark the cases correctly predicted to occur in ALD, and red dots mark the cases correctly predicted to occur in CVD. (b) Flowchart showing the steps to follow when using the developed predictive equation to pre-screen/identify possible head design and operation parameters that likely lead to film growth in ALD.

Figure 4a shows the comparison of the growth regime predicted by the developed predictive equation (regime_predicted_) using a chosen threshold of 0.54 and the growth regime expected based on the different simulation cases (regime_expected_). The figure shows that >90% of the cases expected to lead to depositions in the ALD regime (purple dots) and in the CVD regime (red dots) were correctly classified by the predictive equation (i.e., regime_predicted_ = regime_expected_). Furthermore, Figure 4a also shows how the choice of threshold affects the performance of the predictive equation and how the threshold can be tuned depending on the deposition requirements. For instance, a user wishing to guarantee perfectly uniform depositions done in the ALD regime may want to consider a threshold lower than 0.54. A threshold of, e.g., 0.45 would imply a lower dashed line in Figure 4a, meaning that there would be even fewer conditions that were expected to occur in the CVD regime but incorrectly predicted to occur in the ALD regime (light red dots in Figure 4a under the dashed line). In other words, more of the conditions expected to occur in CVD (red dots in Figure 4a) would also be predicted to occur in CVD, thus being excluded from the list of deposition conditions to be considered. Moreover, a user wishing to deposit in the ALD regime and wanting to find a wide range of conditions to do so may want to consider a higher threshold, e.g., 0.6. Such a higher threshold would raise the dashed line in Figure 4a, meaning that more of the conditions expected to occur in ALD (purple dots in Figure 4a) would be correctly predicted to occur in ALD, thus being included in the list of deposition conditions to be considered by the user. Therefore, the predictive equation developed in this study allows users not only to predict the film growth regime to be expected for a given set of head design parameters and operating conditions but also to modulate the performance of the predictive equation by adjusting the threshold of regime_predicted_ to suit the requirements of the depositions.

Figure 4b shows a flowchart with the steps to follow when using the developed predictive equation to pre-screen/identify possible head design and operation parameters that likely lead to film growth in ALD. Following the flowchart in Figure 4b, the user would initially choose a set of head design and operational parameters, based on the requirements/limitations/preferences for the deposition. For example, because the geometries of the head depend on the method used to fabricate it, one may need to consider a head with walls no thinner than 600 μm (i.e., wall_thick_ = 600 μm). This value together with other initial design and operation parameters would be introduced in eq 4 to produce an initial prediction of the growth regime, i.e., regime_predicted_. A value of regime_predicted_ above a threshold of 0.54 would imply that film growth in the CVD regime would likely occur, and, in that case, the user could simply change one or more of the initially set parameters to decrease the value of regime_predicted_ until it is below the threshold. Once that is achieved, the identified design and operation parameters could be considered to guide the building of the head and the choice of operating conditions during the experiments, as that will likely lead to film growth in the ALD regime. To grow films in the CVD regime instead, one would simply need to identify sets of parameters producing a value of regime_predicted_ above the threshold.

When using the developed predictive equation to pre-screen possible design and operation parameters, it is important to consider how the different parameters affect the precursor intermixing. In this iterative process, the results discussed in Sections 3.1 and 3.2 offer valuable insights into how to change the different parameters to obtain the targeted film growth regime. Furthermore, the coefficients of eq 4 (Table 2) can also be of assistance because they provide information on the relative importance of the different parameters in the equation, over the predicted growth regime (regime_predicted_), with larger coefficients indicating a larger influence over the predicted growth regime. The coefficient of the flow rate ratio (*a*_5_, eq 4) is one order of magnitude larger than the coefficients for all other parameters (*a*_1_–*a*_4_, eq 4), indicating that the flow rate ratio has the largest footprint over the growth regime. The coefficients for all the remaining parameters are within the same order of magnitude, with the deposition gap (*a*_3_, eq 4) having the second largest footprint over the growth regime. This is convenient because these two parameters (flow rate ratio and deposition gap) are much easier to change experimentally than the others (e.g., wall thicknesses and exhaust width). Moreover, the sign of the coefficients shows directly how the corresponding parameters affect the predicted regime, with a positive coefficient indicating that increasing values of the corresponding parameter will lead to increasing values of the predicted regime variable. This information could be used to decide how to change the different parameters of the deposition to obtain a regime_predicted_ below the defined threshold and, thus, identify sets of head design and operation parameters that likely produce film growth in the ALD regime.

Finally, the developed predictive equation can be used to plot the variable regime_predicted_ as a function of the different design and operation parameters (Figures S12 and S13) to enable fine optimization of the different parameters controlling the film growth regime. To simplify this optimization, a spreadsheet with all the plots shown in Figures S12 and S13 was developed and made available (Supporting Information), where all the design and operation parameters can be directly changed, to rapidly predict the film growth regime to be expected for any set of chosen parameters. The abovementioned predictive equation, the supporting graphical elements (e.g., designs maps in Figures 3, S7, and S8 and plots in Figures S12 and S13), and the spreadsheet developed in this work (Supporting Information) offer various convenient and simple ways to pre-screen/identify possible head design and operation parameters to guide the preparation of the depositions.

## 4 Conclusions

We have used numerical simulation to identify how to rationally design and operate SALD systems to grow thin films in ALD or CVD regimes. We have built design maps that show the proportion of the substrate with precursor intermixing for multiple head design and operation parameters and developed a predictive equation for predicting the film growth regime, as a function of the head design and operating conditions. We showed that the film growth regimes predicted by the developed equation are in full agreement with the growth regimes observed in our experiments, demonstrating that the results of the present work can assist users in designing, operating, and optimizing SALD systems for controlled thin-film deposition. This work offers the tools to conveniently screen possible deposition conditions and make quick informed decisions on how to design and operate SALD systems for depositing films in different growth regimes. This is important for controlling the physical and chemical properties of thin films and for applications relying on the precise control over the properties of the deposited materials.

## Supporting Information Available

Data-driven modeling approach, validation, experimental conditions and results, design maps for additional precursor flow rates, receiver operating characteristic curves and the area under those curves for alternative predictive equations, and examples of the application of the developed predictive equation (PDF)
Parameters used in simulations for testing of the predictive equations (TXT)
Implementation of the developed predictive equation whose input parameters and conditions can be readily changed to assess their impact over the predicted values (XLSX)
