# Supplementary material for: Can We Rationally Design and Operate Spatial Atomic Layer Deposition Systems for Steering the Growth Regime of Thin Films?
Source: J Phys Chem C Nanomater Interfaces. 2023 May 5;127(19):9425–36. doi: 10.1021/acs.jpcc.3c02262 (PMC10201529; doi:10.1021/acs.jpcc.3c02262)
Supplement: Supplementary file 1 — jp3c02262_si_001.pdf [file jp3c02262_si_001.pdf]

Can We Rationally Design and Operate Spatial Atomic Layer Deposition Systems for Steering the Growth Regime of Thin Films?

João Pedro Vale,[1,2] Abderrahime Sekkat,[3,4] Thomas Gheorghin,[3] Semih Sevim,[5] Eirini Mavromanolaki,[6] Andreas D Flouris,[7,8] Salvador Pané,[5] David Muñoz Rojas,[3] Josep Puigmartí-Luis,[9,10*] Tiago Sotto Mayor[1,2*]

[1]Transport Phenomena Research Centre (CEFT), Engineering Faculty of Porto University, Rua Dr Roberto Frias, 4200-465 Porto, Portugal

[2]Associate Laboratory in Chemical Engineering (ALiCE), Engineering Faculty of Porto University, Rua Dr Roberto Frias, 4200-465 Porto, Portugal

[3]Université Grenoble Alpes, CNRS, Grenoble INP, LMGP, Grenoble, France

[4]Laboratoire de Génie Chimique, Université de Toulouse, CNRS, Toulouse, France

[5]Multi-Scale Robotics Lab, Institute of Robotics and Intelligent Systems, ETH Zurich, Tannenstrasse 3, CH-8092 Zurich, Switzerland.

[6]Creative Nano PC, Athens, Greece

[7]Discovery Foundation, Heraklion, Crete, Greece

[8]FAME Laboratory, Department of Physical Education and Sport Science, University of Thessaly, Greece

[9]Departament de Ciència dels Materials i Química Física, Institut de Química Teòrica i Computacional, University of Barcelona (UB), 08028 Barcelona, Spain

[10]Institució Catalana de Recerca i Estudis Avançats (ICREA), Pg. Lluís Companys 23, 08010 Barcelona, Spain

E-mail: josep.puigmarti@ub.edu, tiago.sottomayor@fe.up.pt

Supplementary information

Data-driven modelling to develop predictive equation of film growth regime

Following a data-driven modelling approach, we used the ordinary least-squares method[1] to develop an equation that can predict the film growth regime of a deposition, depending on the head design and operation parameters of spatial atomic layer deposition systems, based on the data obtained from the 2700 simulation cases considered in this work (Table 1).

The ordinary least-squares method is a technique to estimate coefficients of linear regression equations describing the relationship between a quantitative independent variable and the dependent variables. This is done by minimizing the sum of the squared residuals between the actual and predicted values. For example, using a general dataset containing the independent variable y_i and a dependent variable x_i, the least-squares method can be used to develop an equation that predicts y_i by calculating its predicted values $\hat{y_i}$ computed as a function of the dependent variable x_i:

$$\hat{y_i} = a_1 x_i + a_0 \tag{1}$$

In this equation, the coefficients a_1 and a_0 are fitted to minimize the sum of the squared residuals (where residual = $y_i - \hat{y_i}$) so that the deviation between the predicted value $\hat{y_i}$ and the actual value y_i is minimal. Then, the predicted value of y_i (i.e. $\hat{y_i}$) can be calculated for different values of the dependent variable x_i, with a_1 informing on how a change in x_i may affect y_i. The coefficients of equation 1 can be calculated by:

$$a_1 = \frac{\sum_{i=1}^{n}(x_i - \bar{x})(y_i - \bar{y})}{\sum_{i=1}^{n}(x_i - \bar{x})^2} \tag{2}$$

$$a_0 = \bar{y} - a_1\bar{x} \tag{3}$$

where $\bar{x}$ and $\bar{y}$ are the mean values of x_i and y_i respectively.

We considered predictive equations with order between 0.5 and 2, including square root, linear and quadratic terms, as well as terms with the product of different parameters (e.g. $wall_{thick} \times dep_{gap}$). To

prevent overfitting, we excluded terms with p-values > 0.05 and parameters that caused collinearity if the condition number was above 10^3 (collinearity found by plotting the correlation matrices).[2]

Using a dummy variable $regime_{expected}$, the simulation cases were classified as depositions in the ALD regime when the proportion of substrate with intermixing was 0 % in the numerical simulations (i.e. $regime_{expected}$ = ALD, coded as 0 in the binary classification), or as depositions in the CVD regime when the proportion of substrate with intermixing was > 0 % in the numerical simulations (i.e. $regime_{expected}$ = CVD, coded as 1 in the binary classification). We then fitted the mentioned predictive equations with different orders, to enable calculating a variable representing the predicted regime ($regime_{predicted}$) as a function of the values of the different parameters (i.e. wall thickness, exhaust width, deposition gap, precursor flow rate and flow rate ratio). For each predictive equation, we defined a threshold for the variable $regime_{predicted}$ below which the depositions were predicted to occur in ALD ($regime_{predicted}$ < threshold), and above which the depositions were predicted to occur in CVD ($regime_{predicted}$ > threshold). The threshold values were chosen to ensure most of the cases in the ALD regime were correctly classified, while only ≈10% of the cases in the CVD regime were incorrectly classified.

For each predictive equation (and threshold), we assessed the performance of the predictive equations by calculating their accuracy and precision,

$$accuracy = \frac{(TP + TN)}{(TP + FN + TN + FP)} \tag{4}$$

$$precision = \frac{TP}{(TP + FP)} \tag{5}$$

where accuracy refers to the percentage of cases correctly predicted to correspond to depositions in ALD or CVD, and precision refers to the percentage of cases correctly predicted to correspond to depositions in ALD in relation to those correctly and incorrectly predicted to correspond to depositions in ALD. In the above equations, TP stands for true positives, FP stands for false positives, TN stands for true negatives and FN stands for false negatives (Table S1). In the context of this work, true positives are cases that were predicted to correspond to ALD by the predictive equations (i.e. $regime_{predicted}$ < threshold) and the numerical simulations (i.e. $regime_{expected}$ = ALD). False positives are cases that were

predicted to correspond to ALD by the predictive equations and expected to correspond to CVD based on the numerical simulations (i.e. $regime_{expected}$ = CVD). True negatives are cases that were predicted to correspond to CVD by the predictive equations (i.e. $regime_{predicted}$ > threshold) and expected to correspond to CVD based on the numerical simulations. Finally, false negatives are cases that were predicted to correspond to CVD by the predictive equations and expected to correspond to ALD based on the numerical simulations. Note that, to reduce bias, the performance of the different predictive equations was assessed using numerical simulation data that was not used to develop them. The performance was assessed considering an extra set of 540 numerical simulations, i.e. 20% of the simulation cases used to develop the predictive equations. In these extra simulation cases, each parameter was assigned a random value within the ranges shown in Table 1 (see parameters in the Supplementary data files).

When choosing the thresholds for the various predictive equations, we considered the corresponding receiver operating characteristic curves[2] (ROC curves; Figure S10a), generated for increasing values of thresholds. The ROC curves, which relate the true positive rate (TP / (TP + FN)) with the false positive rate (FP / (FP + TN)), are useful because they allow to directly compare different predictive equations, knowing that a higher area under the ROC curve (AUC) is indicative of a better classifier (an AUC of 1 corresponds to a perfect classifier). Furthermore, the ROC curves allow to find threshold values leading to high true positive rates and low false positive rates, therefore leading to high values of accuracy and precision of the predictive equations.

The ROC curves in Figure S10a show that predictive equation with order 0.5 (equation 4, in the main manuscript) is the best classifier for the data in this work, because its ROC curve has the highest area under the curve (AUC = 0.98). Moreover, that the predictive equation with order 0.5 is the best classifier is further confirmed by the fact that it has the highest accuracy (≈ 93 %, Figure S10b-d), with a precision similar to that of the other predictive equations (≈ 96 %). The predictive equation with order 0.5 was also shown to have the highest performance when the same threshold (e.g. 0.5) was used for the three predictive equations (Figure S11). For these reasons, a predictive equation with order 0.5 (equation 4 in the main manuscript) was adopted in this work to classify the film growth regime.

The coefficients (values and standard errors) of the various predictive equations considered in this work are shown in Tables 2, S6 and S7. The mentioned coefficients were calculated considering the units of the parameters as shown in Table 1, i.e. wall thickness, exhaust width and deposition gap in μm, and precursor flow rate in sccm (flow rate ratio being dimensionless). Different units can be used if the coefficients are adjusted accordingly.

Table S1. Representation of the confusion matrix in the context of the present data-driven modelling approach.

		$regime_{predicted}$	
		ALD	CVD
$regime_{expected}$	ALD	true positive (TP)	false negative (FN)
	CVD	false positive (FP)	true negative (TN)

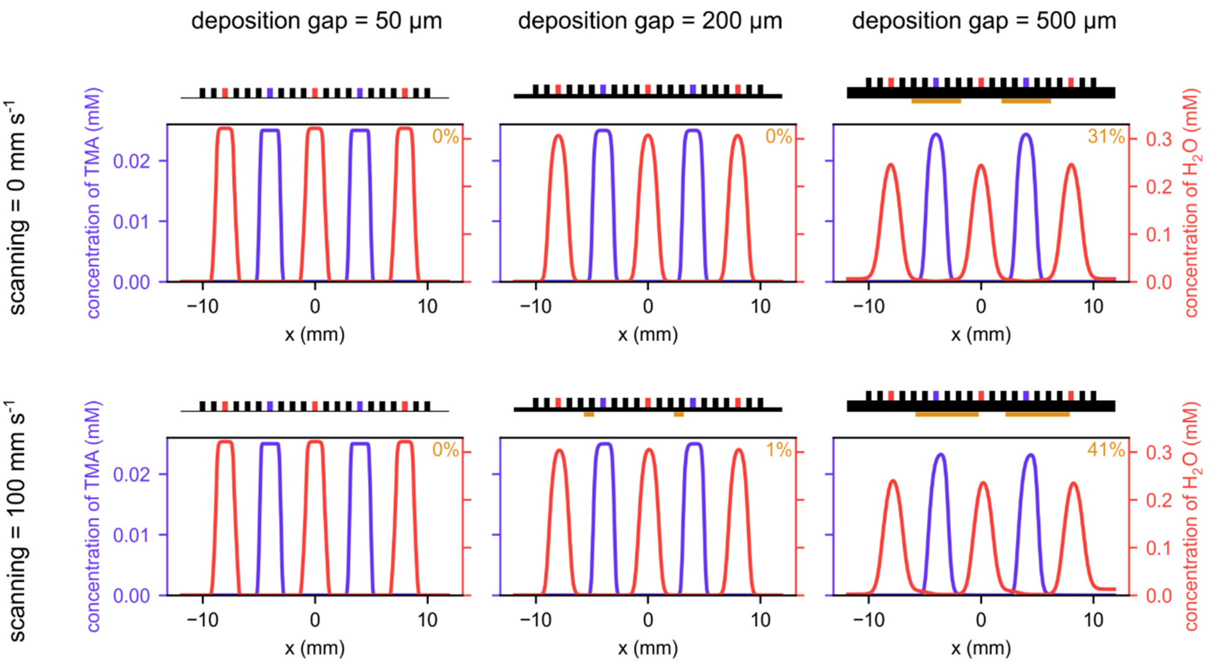

Figure S1. Concentration profile of TMA and H_2O along the substrate (x-axis) while varying deposition gap (50, 200 and 500 µm) and scanning speed (0 and 100 mm·s^{-1}), considering a wall thickness of 500 µm, exhaust width of 500 µm, precursor flow rate of 150 sccm and flow rate ratio of 2. Illustrations of the deposition heads of each simulation case are shown above each plot together with orange lines marking where precursor intermixing is expected. In each simulation case, the orange areas under the concentration profiles mark the regions with precursor intermixing, and the specific proportion of substrate with precursor intermixing is shown in orange at the plots' top-right corner. The substrate motion causes a slight increase in the proportion of substrate with precursor intermixing for the simulations with larger depositions gaps (200 and 500 µm). The effect of scanning grows in importance with increasing deposition gap because of the associated decreasing linear velocity of fluids in the gap (i.e. because of the decreasing difference between the velocity of the fluids and that of the substrate).

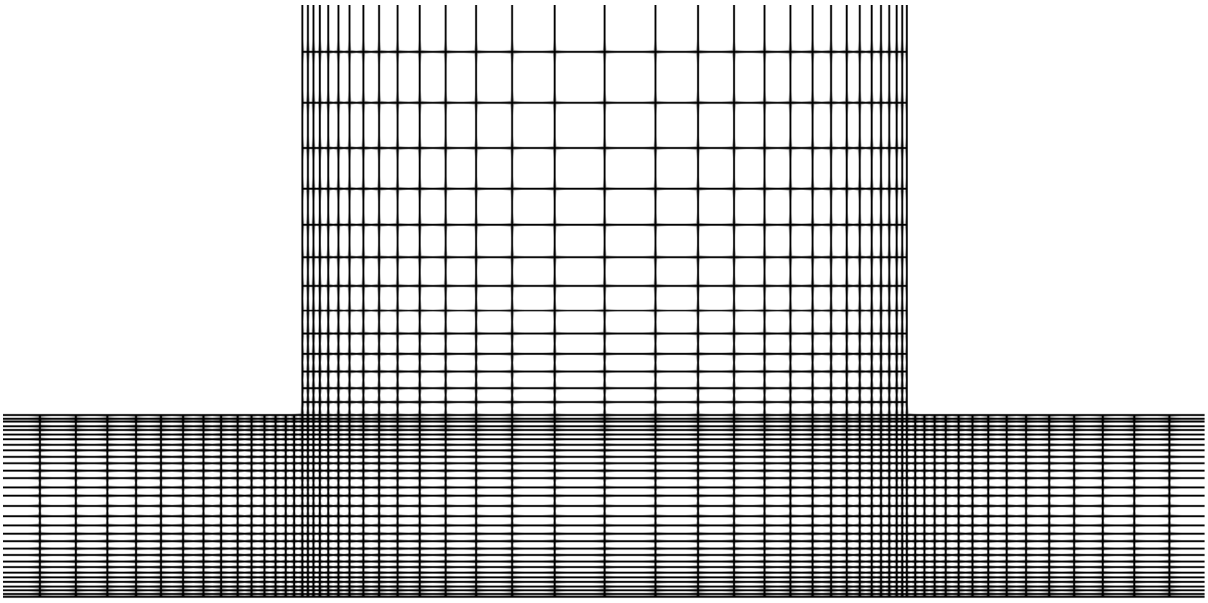

Figure S2. Representation of the mesh used in the simulations, containing a total cell count of 6 x 10^4; The figure shows the cell distribution near a precursor channel, with similar distributions being considered for the other channels.

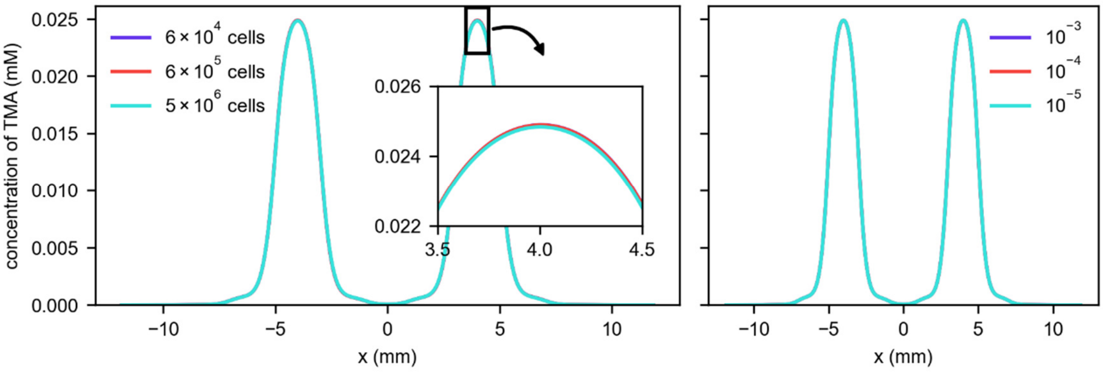

Figure S3. Concentration of TMA along x for meshes with different cell count (6 x 10^4, 6 x 10^5 and 5 x 10^6, left plot) and for different convergence criteria (10^{-3}, 10^{-4}, 10^{-5}, right plot). The deposition head that was considered in mesh size and convergence criteria testing had a channel width of 500 µm, wall thickness of 500 µm, exhaust width of 500 µm, deposition gap of 500 µm, precursor flow rate of 150 sccm and flow rate ratio of 1.

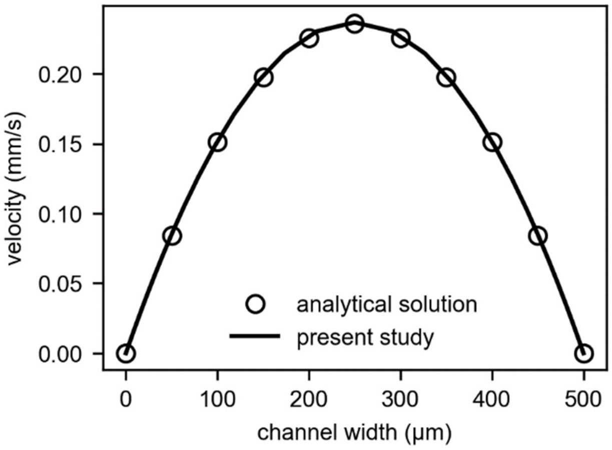

Figure S4. Validation of the velocity profile in the precursor channel by comparing the results obtained in the present study to those obtained with an analytical solution,[3] for a precursor flow rate of 150 sccm at 200 °C.

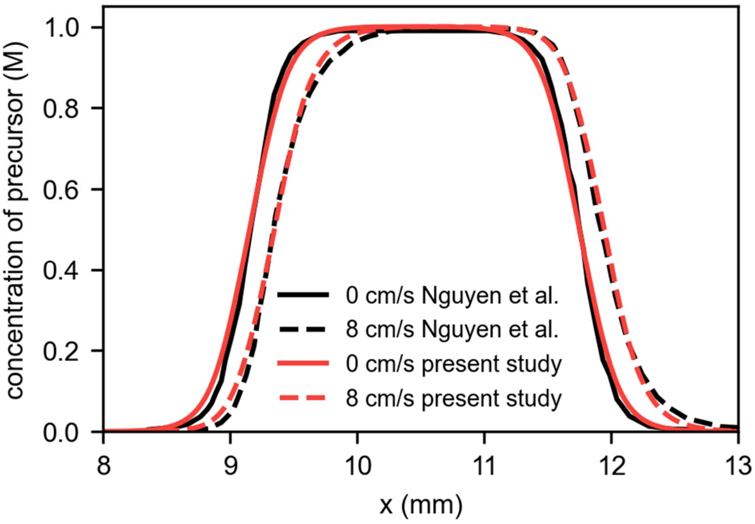

Figure S5. Validation of the concentration of precursor along the substrate by replicating the numerical work by Nguyen et al., 2021[4] for a scanning speed of 0 cm/s and 8 cm/s. The substrate is moving towards the positive x-axis direction.

Table S2. Depositions performed in the present study (#1-#4) and data from the literature (#5-#8) which were used to validate the present model by comparison of actual and the predicted film growth regime. The deposition head that was used in depositions #1-8 had a channel width of 500 µm, wall thickness of 800 µm and exhaust width of 500 µm.

#	type of data	T_{sub} (°C)	deposition gap (µm)	flow rate (sccm)			actual result	predicted result	data from
				TMA	H_2O	N_2			
1	Experimental	200	90	125	125	125	ALD	ALD	Present study
2			300				CVD	CVD	
3			30				ALD	ALD	
4			90			250	ALD	ALD	
5	Numerical	150	100	150	150	150	ALD	ALD	Alshehri, 2021[5]
6			230				CVD	CVD	
7	Experimental	150	150	125	125	125	CVD	CVD	
8			230				CVD	CVD	

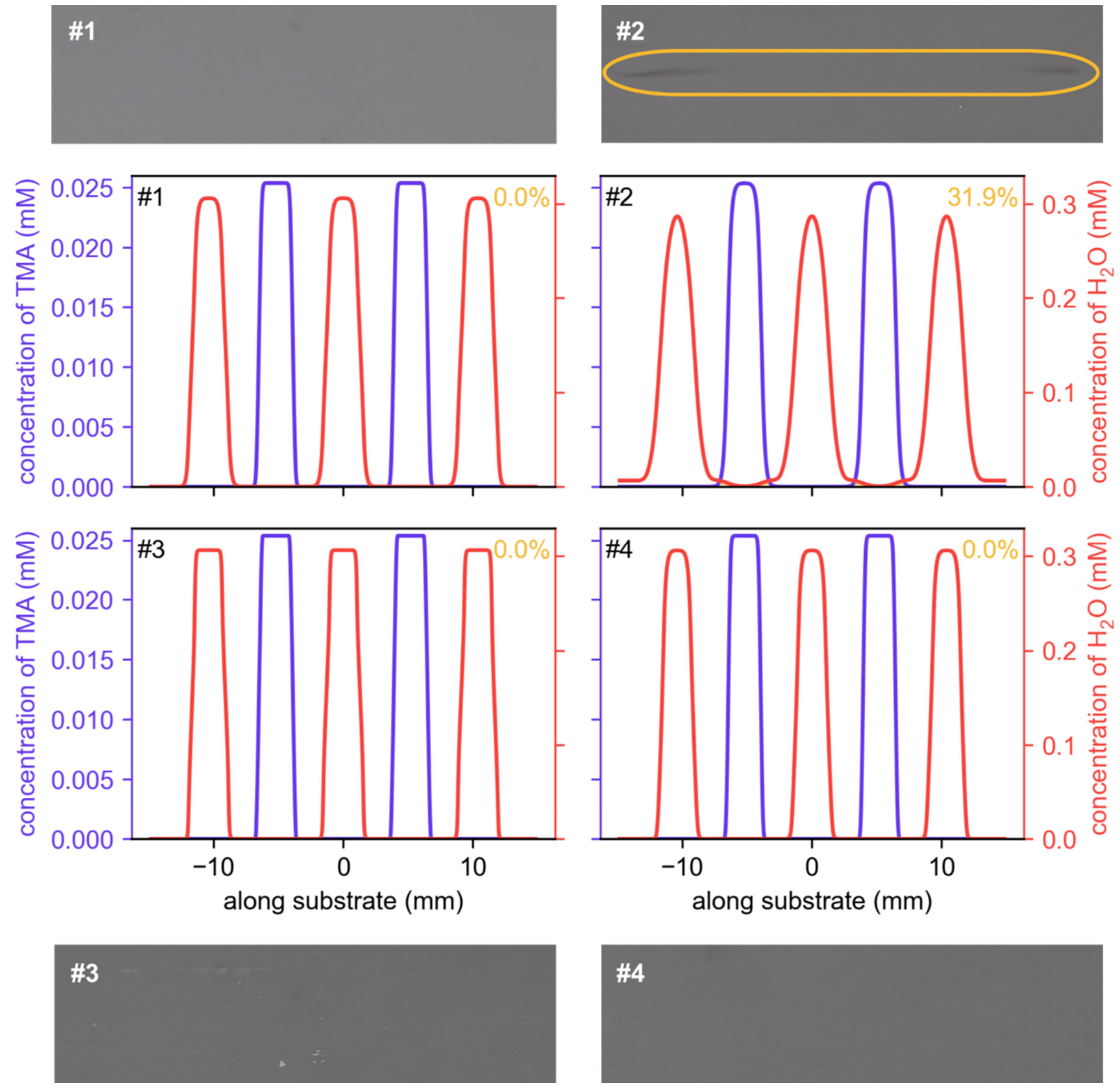

Figure S6. Depositions performed in the present study (grey images marked with #1-#4) and respective concentration profiles of TMA and H_2O that accurately identify the conditions originating film growth in the CVD regime (yellow). The deposition images (#1-#4) show the substrate region under the deposition head. The depositions were performed in static mode, and because that prevents the full ALD cycle, film growth is only visible when the conditions are compatible with CVD regime (#2). Experiments #1 to #3 were performed using a flow rate ratio of 1 and a deposition gap of 90 µm, 300 µm and 30 µm, respectively, while experiment #4 was performed using a flow rate ratio of 2 and a deposition gap of 90 µm (Table S2).

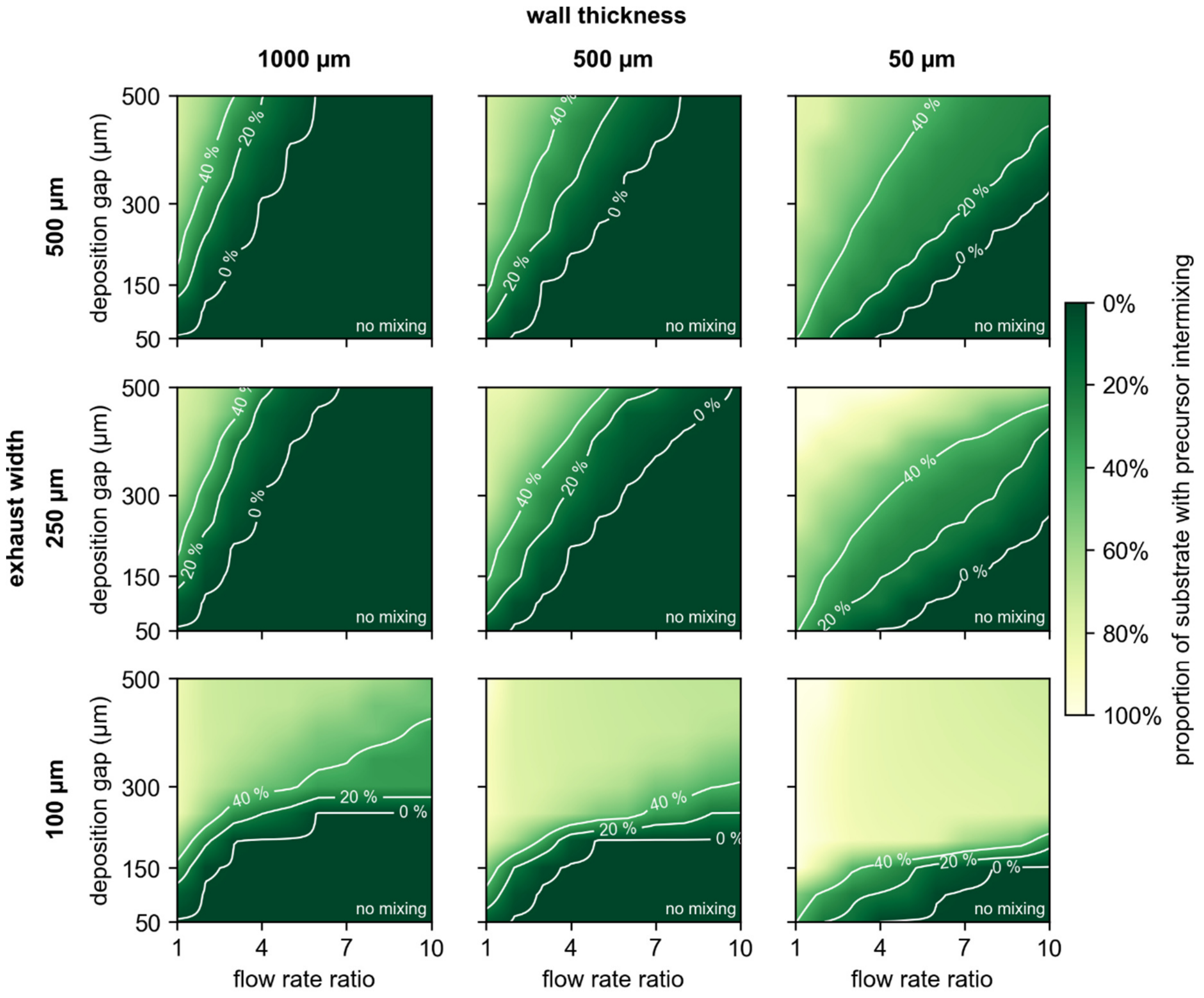

Figure S7. Design maps of proportion of substrate with precursor intermixing as a function of deposition gap and flow rate ratio, for wall thicknesses of 1000, 500 and 50 µm (columns) and exhaust widths of 500, 250 and 100 µm (rows), for a precursor flow rate of 75 sccm. White lines in the maps identify the conditions of equal precursor intermixing proportion. The region below the 0% line represents the conditions that lead to null precursor mixing and, thus, film growth by ALD. The region above the 0% line represents the conditions that lead to some precursor mixing and, thus, film growth by CVD. The specific value of precursor intermixing proportion indicates the proportion of substrate length that is exposed to film growth by CVD. The design maps in this figure show the results of 900 different simulation cases.

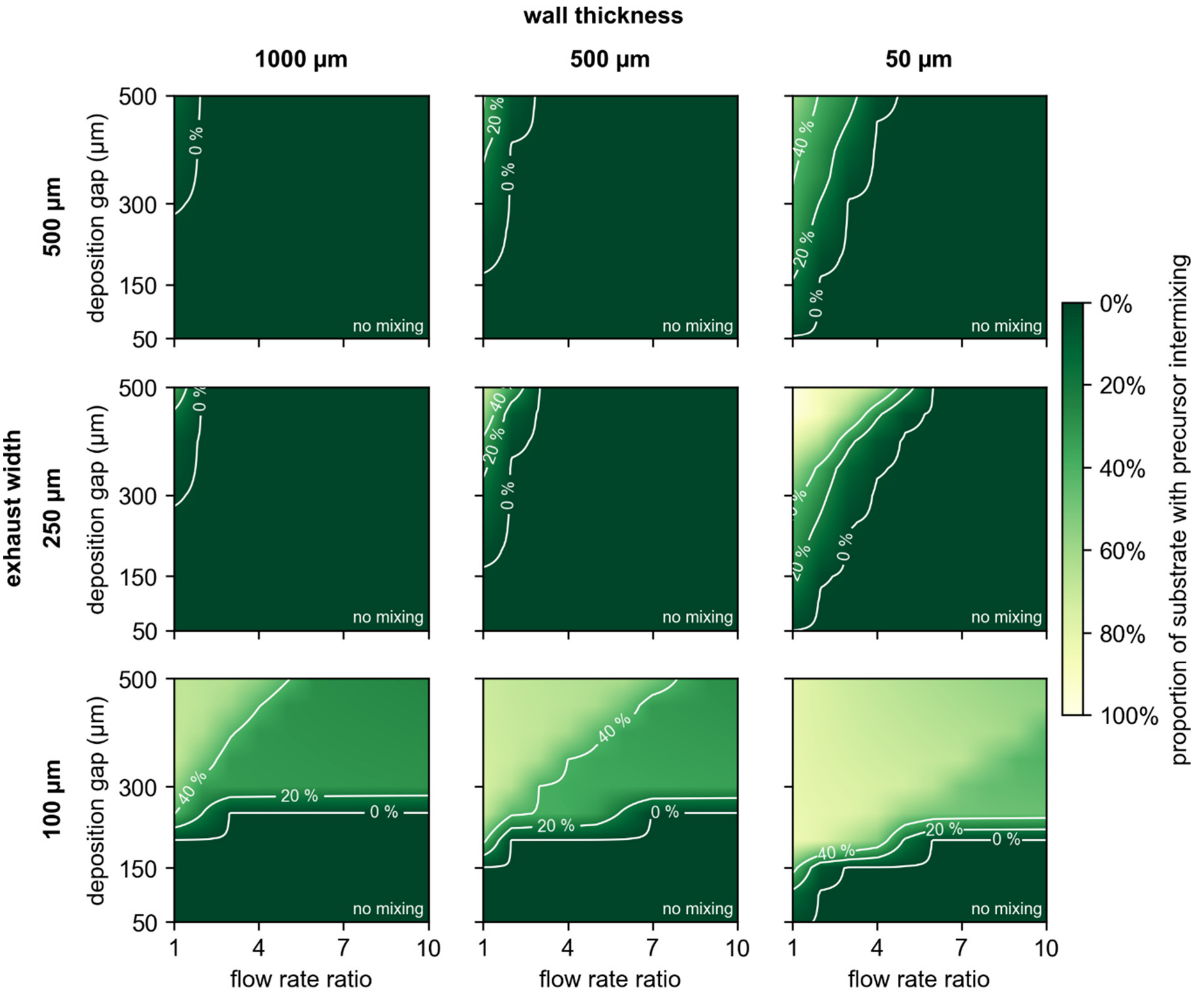

Figure S8. Design maps of proportion of substrate with precursor intermixing as a function of deposition gap and flow rate ratio, for wall thicknesses of 1000, 500 and 50 µm (columns) and exhaust widths of 500, 250 and 100 µm (rows), for a precursor flow rate of 300 sccm. White lines in the maps identify the conditions of equal precursor intermixing proportion. The region below the 0% line represents the conditions that lead to null precursor mixing and, thus, film growth by ALD. The region above the 0% line represents the conditions that lead to some precursor mixing and, thus, film growth by CVD. The specific value of precursor intermixing proportion indicates the proportion of substrate length that is exposed to film growth by CVD. The design maps in this figure show the results of 900 different simulation cases.

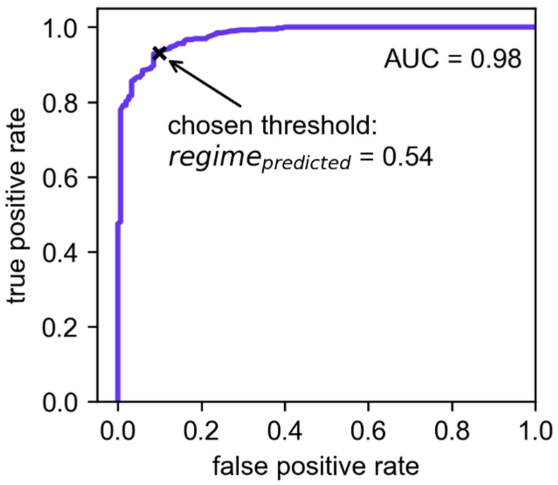

Figure S9. ROC curve of the predictive equation of order 0.5 that was used in the present study. The area under the curve (AUC) and the chosen threshold are represented.

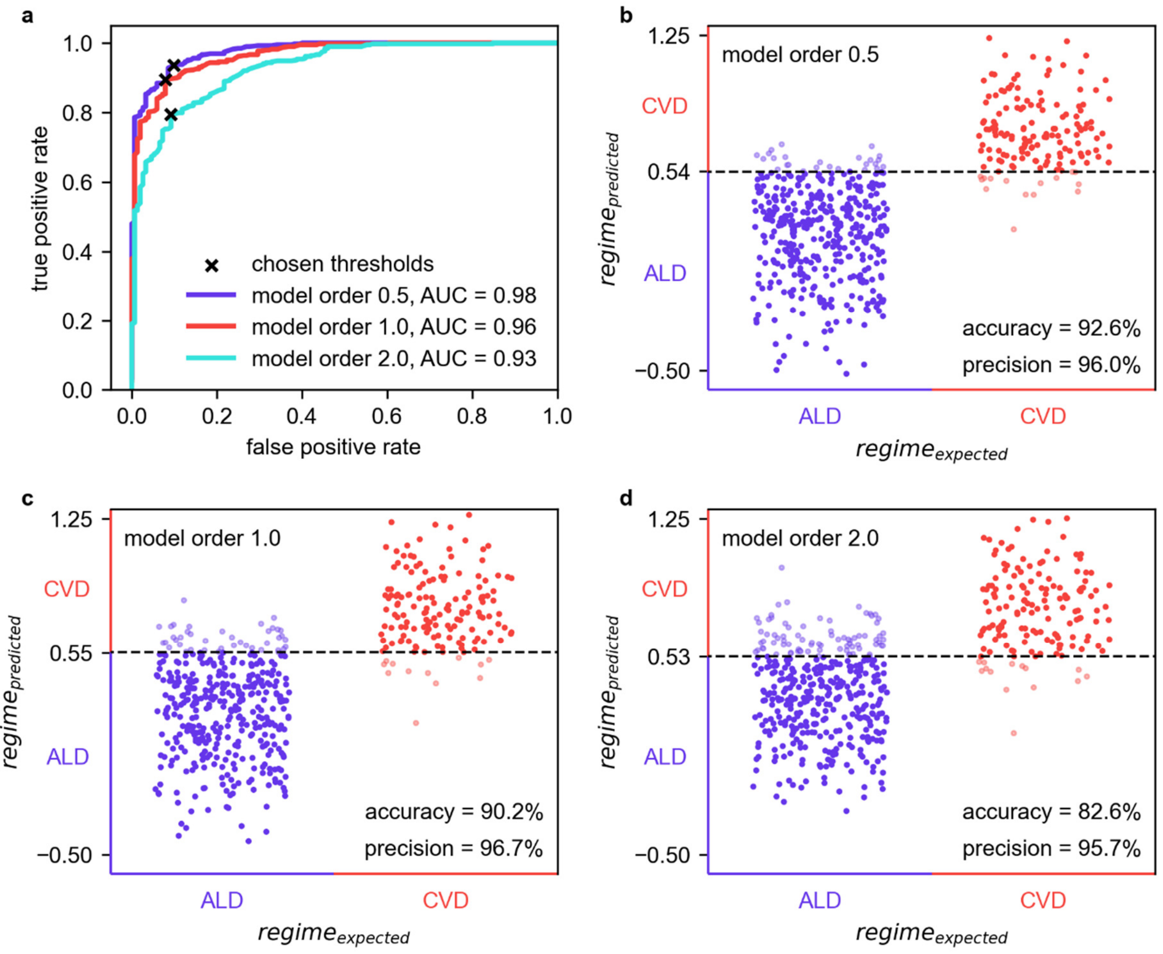

Figure S10. (a) ROC curve of the different predictive equations tested in the present study and their area under the curve (AUC); (b)-(d) Comparison between the prediction given by the predictive equations (*regime*$_{prediction}$) and the expected result obtained by numerical simulation (*regime*$_{expected}$) for the 540 simulations that were performed for testing. Cases below the dashed lines, which represents the chosen thresholds, are predicted to occur in the ALD regime.

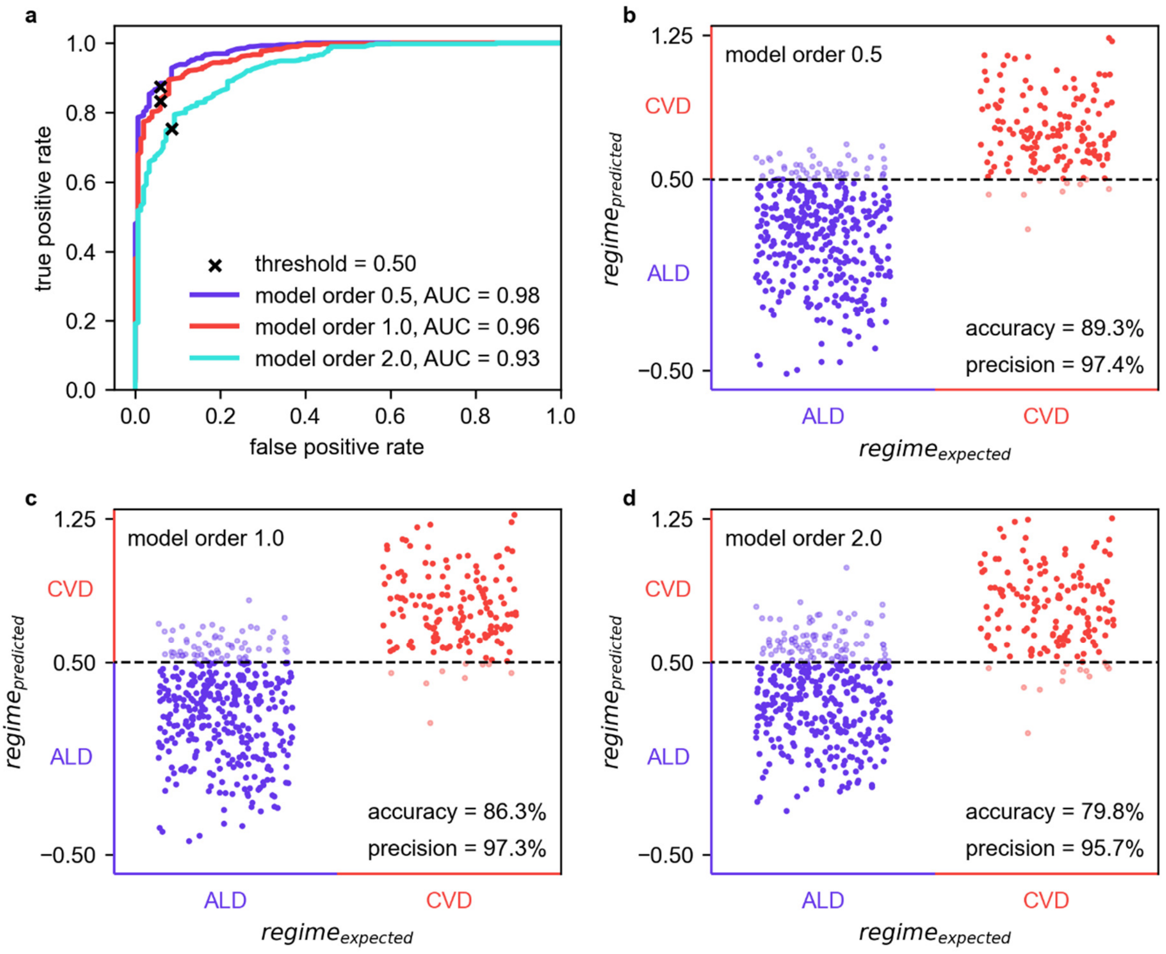

Figure S11. (a) ROC curve of the different predictive equations tested in the present study and their area under the curve (AUC); (b)-(d) Comparison between the prediction given by the predictive equations ($regime_{prediction}$) and the expected result obtained by numerical simulation ($regime_{expected}$) for the 540 simulations that were performed for testing. Cases below the dashed lines, which represents a threshold of 0.5, are predicted to occur in the ALD regime.

Table S3. Confusion matrix showing the performance of the predictive equation of order 0.5 used in the present study.

		$regime_{predicted}$	
		ALD	CVD
$regime_{expected}$	ALD	TP = 363	FN = 25
	CVD	FP = 15	TN = 137

Table S4. Confusion matrix showing the performance of the predictive equation of order 1.

		$regime_{predicted}$	
		ALD	CVD
$regime_{expected}$	ALD	TP = 347	FN = 41
	CVD	FP = 12	TN = 140

Table S5. Confusion matrix showing the performance of the predictive equation of order 2.

		$regime_{predicted}$	
		ALD	CVD
$regime_{expected}$	ALD	TP = 308	FN = 80
	CVD	FP = 14	TN = 138

Table S6. Values and standard errors for each coefficient in the predictive equation of order 1.

parameter	coefficient	value	standard error
$wall_{thick}$	a_1	-3.22 × 10^{-4}	1.68 × 10^{-5}
exh_{width}	a_2	-8.42 × 10^{-4}	3.94 × 10^{-5}
dep_{gap}	a_3	1.50 × 10^{-3}	4.53 × 10^{-5}
pfr	a_4	-1.24 × 10^{-3}	6.95 × 10^{-5}
frr	a_5	-6.64 × 10^{-2}	2.26 × 10^{-3}
1	a_6	1.00	0.03

Table S7. Values and standard errors for each coefficient in the predictive equation of order 2.

parameter	coefficient	value	standard error
${wall_{thick}}^2$	a_1	-2.66 × 10^{-7}	1.67 × 10^{-8}
${exh_{width}}^2$	a_2	-1.19 × 10^{-6}	6.89 × 10^{-8}
${dep_{gap}}^2$	a_3	2.48 × 10^{-6}	8.76 × 10^{-8}
pfr^2	a_4	-3.00 × 10^{-6}	1.95 × 10^{-7}
frr^2	a_5	-5.26 × 10^{-3}	2.19 × 10^{-4}
1	a_6	0.75	0.02

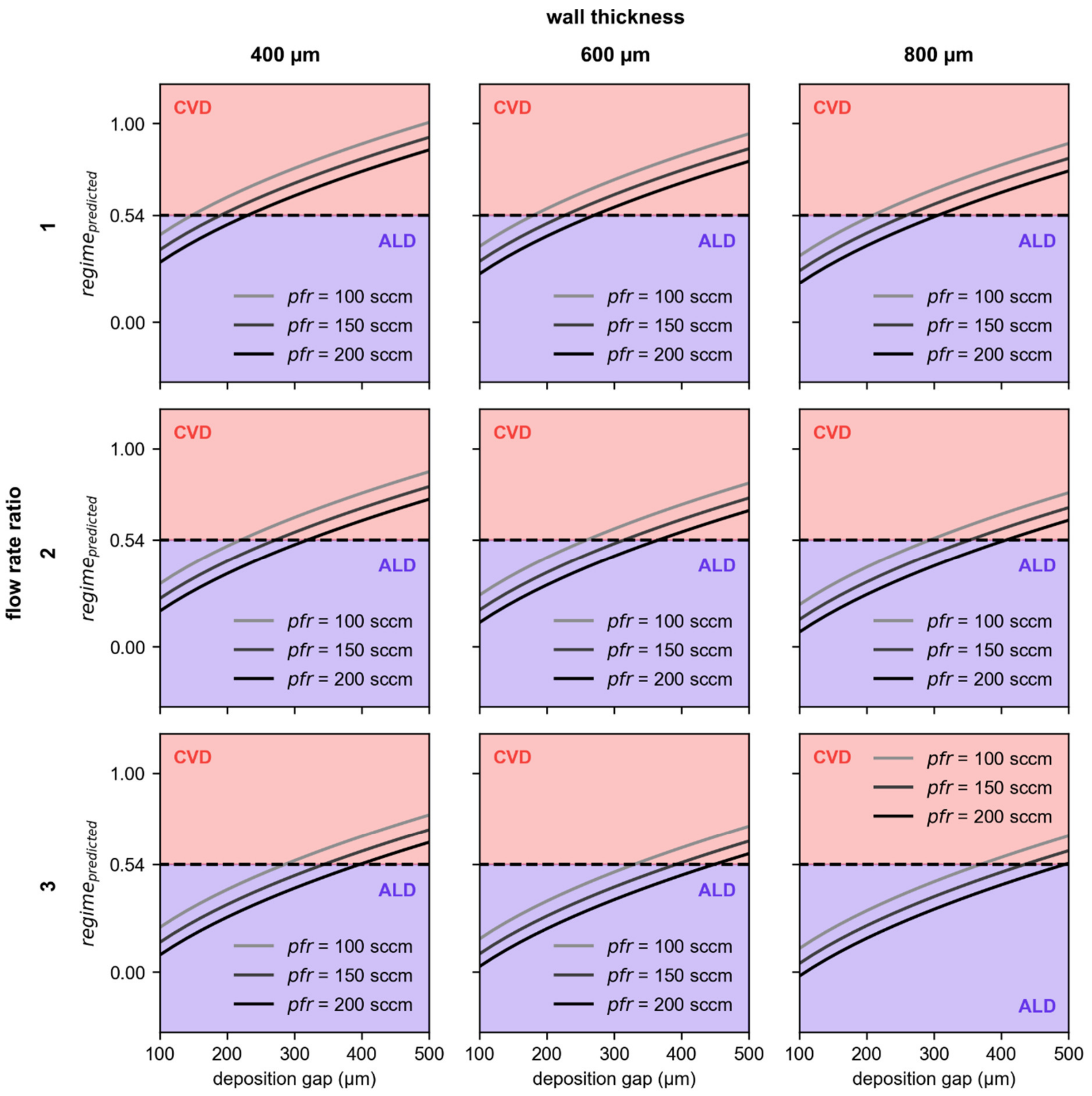

Figure S12. Growth regime predicted by the predictive equation developed in the present study, for various wall thicknesses, flow rate ratios, precursor flow rates and deposition gaps (for an exhaust width of 500 µm). Conditions below the dashed line (i.e. in the blue region, which corresponds to a $regime_{predicted}$ < 0.54) likely lead to film growth in ALD.

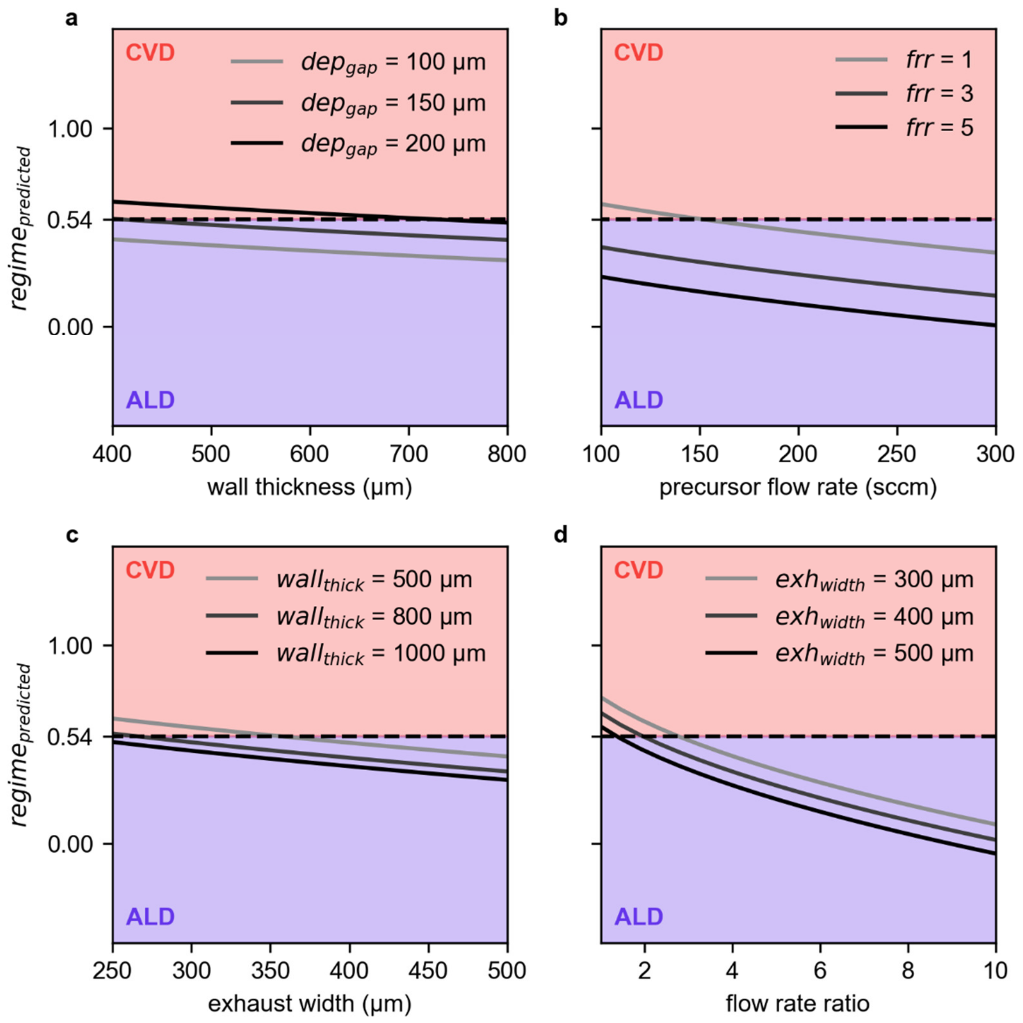

Figure S13. Growth regime predicted by the predictive equation developed in the present study, for various conditions: (a) increasing wall thicknesses and deposition gaps, for exhaust width = 500 µm, precursor flow rate = 100 sccm, and flow rate ratio = 1; (b) increasing precursor flow rates and flow rate ratios, for wall thickness = 200 µm, exhaust width = 500 µm, and deposition gap = 150 µm; (c) increasing exhaust widths and wall thicknesses, for deposition gap = 150 µm, precursor flow rate = 150 sccm, and flow rate ratio = 1; (d) increasing flow rate ratios and exhaust widths, for wall thickness = 300 µm, deposition gap = 200 µm, precursor flow rate = 150 sccm. Conditions below the dashed line (i.e. in the blue region, which corresponds to a $regime_{predicted}$ < 0.54) likely lead to film growth in ALD.

References

(1) Freund, R. J.; Wilson, W. J. *Statistical Methods*; Elsevier, 2010. https://doi.org/10.1016/C2009-0-20216-9.

(2) James, G.; Witten, D.; Hastie, T.; Tibshirani, R. *An Introduction to Statistical Learning*; Springer Texts in Statistics; Springer New York: New York, NY, 2013; Vol. 103. https://doi.org/10.1007/978-1-4614-7138-7.

(3) White, F. *Viscous Fluid Flow*; McGraw-Hill: New York, 1991.

(4) Nguyen, V. H.; Sekkat, A.; Jiménez, C.; Muñoz, D.; Bellet, D.; Muñoz-Rojas, D. Impact of Precursor Exposure on Process Efficiency and Film Properties in Spatial Atomic Layer Deposition. *Chem. Eng. J.* **2021**, *403* (April 2020), 126234. https://doi.org/10.1016/j.cej.2020.126234.

(5) Alshehri, A. H.; Loke, J. Y.; Nguyen, V. H.; Jones, A.; Asgarimoghaddam, H.; Delumeau, L. V.; Shahin, A.; Ibrahim, K. H.; Mistry, K.; Yavuz, M.; Muñoz-Rojas, D.; Musselman, K. P. Nanoscale Film Thickness Gradients Printed in Open Air by Spatially Varying Chemical Vapor Deposition. *Adv. Funct. Mater.* **2021**, *2103271* (31), 1–14. https://doi.org/10.1002/adfm.202103271.
